# Translational implications of Th17-skewed inflammation due to genetic deficiency of a cadherin stress sensor

**DOI:** 10.1172/JCI144363

**Published:** 2022-02-01

**Authors:** Lisa M. Godsel, Quinn R. Roth-Carter, Jennifer L. Koetsier, Lam C. Tsoi, Amber L. Huffine, Joshua A. Broussard, Gillian N. Fitz, Sarah M. Lloyd, Junghun Kweon, Hope E. Burks, Marihan Hegazy, Saki Amagai, Paul W. Harms, Xianying Xing, Joseph Kirma, Jodi L. Johnson, Gloria Urciuoli, Lynn T. Doglio, William R. Swindell, Rajeshwar Awatramani, Eli Sprecher, Xiaomin Bao, Eran Cohen-Barak, Caterina Missero, Johann E. Gudjonsson, Kathleen J. Green

**Affiliations:** 1 Department of Pathology and; 2 Department of Dermatology, Feinberg School of Medicine, Northwestern University, Chicago, Illinois, USA.; 3 Department of Dermatology,; 4 Department of Computational Medicine & Bioinformatics, and; 5 Department of Biostatistics, University of Michigan, Ann Arbor, Michigan, USA.; 6 Robert H. Lurie Comprehensive Cancer Center, Northwestern University, Chicago, Illinois, USA.; 7 Department of Molecular Biosciences, Northwestern University, Evanston, Illinois, USA.; 8 Department of Pathology, University of Michigan, Ann Arbor, Michigan, USA.; 9 CEINGE Biotecnologie Avanzate, Naples, Italy.; 10 Department of Pharmacology, Feinberg School of Medicine, Northwestern University, Chicago, Illinois, USA.; 11 Department of Internal Medicine, The Jewish Hospital, Cincinnati, Ohio, USA.; 12 Department of Neurology, Feinberg School of Medicine, Northwestern University, Chicago, Illinois, USA.; 13 Department of Dermatology, Tel Aviv Medical Center, Department of Human Molecular Genetics & Biochemistry, Sackler Faculty of Medicine, Tel Aviv University, Tel Aviv, Israel.; 14 Department of Dermatology, “Emek” Medical Center, Afula, Israel.; 15 Rappaport Faculty of Medicine, Technion, Haifa, Israel.; 16 Department of Biology, University of Naples, Naples, Italy.

**Keywords:** Dermatology, Immunology, Bioinformatics, Genetic diseases, Skin

## Abstract

Desmoglein 1 (Dsg1) is a cadherin restricted to stratified tissues of terrestrial vertebrates, which serve as essential physical and immune barriers. Dsg1 loss-of-function mutations in humans result in skin lesions and multiple allergies, and isolated patient keratinocytes exhibit increased proallergic cytokine expression. However, the mechanism by which genetic deficiency of Dsg1 causes chronic inflammation is unknown. To determine the systemic response to Dsg1 loss, we deleted the 3 tandem Dsg1 genes in mice. Whole transcriptome analysis of embryonic *Dsg1^–/–^* skin showed a delay in expression of adhesion/differentiation/keratinization genes at E17.5, a subset of which recovered or increased by E18.5. Comparing epidermal transcriptomes from Dsg1-deficient mice and humans revealed a shared IL-17–skewed inflammatory signature. Although the impaired intercellular adhesion observed in *Dsg1^–/–^* mice resembles that resulting from anti-Dsg1 pemphigus foliaceus antibodies, pemphigus skin lesions exhibit a weaker IL-17 signature. Consistent with the clinical importance of these findings, treatment of 2 Dsg1-deficient patients with an IL-12/IL-23 antagonist originally developed for psoriasis resulted in improvement of skin lesions. Thus, beyond impairing the physical barrier, loss of Dsg1 function through gene mutation results in a psoriatic-like inflammatory signature before birth, and treatment with a targeted therapy significantly improved skin lesions in patients.

## Graphical abstract

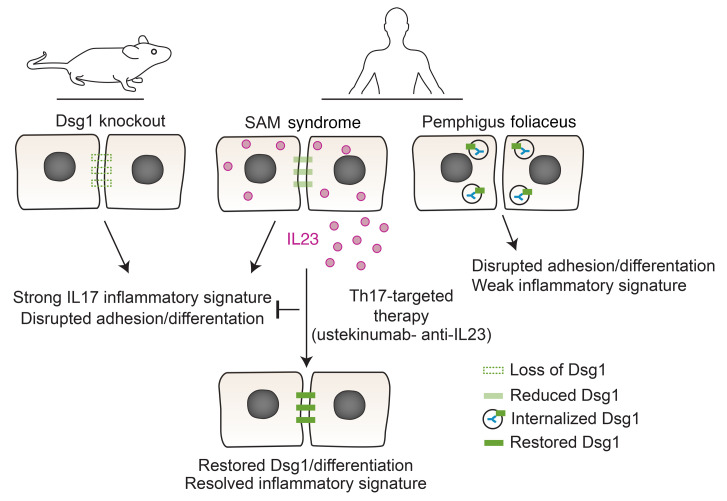

## Introduction

The multilayered epidermal barrier is made up of interdependent microbiome, chemical, physical, and immune components. These components work together to protect against water loss, physical insults, and infection (1, 2). The asymmetric distribution of membrane proteins along the apical to basal axis of simple epithelia ensures that epithelial barrier and transport functions are properly regulated. However, in multilayered epithelia such as the epidermis, architectural features are polarized along the entire apical to basal axis of the stratified epithelium. Disorganization or loss of these polarized features disrupts barrier function of the epidermis and causes cutaneous disease (3).

Among the most polarized molecules in the epidermis are desmosomal cadherins, desmogleins (Dsgs), and desmocollins (Dscs) (4, 5). These transmembrane cell-cell adhesion molecules cooperate with plakins and armadillo proteins to anchor intermediate filaments (IFs) to the desmosome, providing tissues with tensile strength. Mutations, bacterial toxins, pemphigus autoimmune antibodies, and dysregulated expression of these cadherins cause a range of mild to potentially lethal disorders including keratodermas, blistering diseases, and cancer in humans and mouse models (5–9).

Among the recently identified disorders caused by mutations in desmosome molecules is severe dermatitis, multiple allergies, and metabolic wasting (SAM) syndrome (10–15). This disorder was initially identified in patients harboring biallelic mutations in desmoglein 1 (Dsg1), resulting in a reduction of expression and/or failure to accumulate at the plasma membrane. Patients with SAM syndrome exhibit abnormal epidermal differentiation, recurrent skin infections, and severe allergies. While the loss of barrier-forming roles of Dsg1 may contribute to these symptoms, isolated patient keratinocytes exhibit cell-autonomous production of cytokine mRNAs, including the proallergic cytokines thymic stromal lymphopoietin (*TSLP*), *IL-5*, and tumor necrosis factor (*TNF*) (10, 14). Further, knockdown of Dsg1 in normal human keratinocytes in vitro was sufficient to induce the gene expression of proinflammatory cytokines *IL-1B*, *IL-6*, *IL-8*, *CXCL1*, and *TNF* (14, 16). Combined with the finding that Dsg1 is downregulated in response to environmental stresses, such as UV exposure, these results raise the possibility that Dsg1 is a stress sensor that governs the expression of cytokines independently of its roles in maintaining tissue integrity and barrier function (10, 16–18).

Functions for desmosomal cadherins that transcend adhesive roles have also recently emerged. For instance, Dsg1 promotes keratinocyte differentiation through attenuation of Ras-Raf signaling to Erk, which requires Dsg1 binding to ErbB2 interacting protein (Erbin) (19, 20). Attenuation of this pathway by Dsg1 facilitates differentiation without inhibiting basal cell proliferation. In addition to its roles in harnessing Erk signaling, we showed that Dsg1 remodels the cortical actin cytoskeleton to temporarily reduce tension in basal cells, which is necessary for promoting stratification in human epidermal organotypic cultures (21). Thus, Dsg1 coordinates a transcriptional and mechanical program of epidermal differentiation and morphogenesis in an in vitro human model. Progress in elucidating adhesion-dependent and -independent functions of Dsg1 in vivo has been hampered by the lack of a fully characterized animal model.

Here we report results from an animal model in which the tandemly arrayed *Dsg1 a, b*, and *c* genes were removed using CRISPR/Cas9-mediated gene editing. Like a similar recently described model, we observed severe adhesion defects and skin peeling in Dsg1-deficient animals (22). Comparisons between the transcriptome profiles of the embryonic skin of Dsg1-deficient animals and skin from patients with SAM syndrome revealed shared changes in keratinization, cornified envelope formation, keratinocyte differentiation, skin development pathways, and an increase in inflammatory response pathways. We compared these data sets with patient cohorts from 2 common inflammatory disorders: atopic dermatitis (AD), largely a Th2-dependent disorder, and psoriasis (PSO), largely a Th17-dependent disorder (23). The previously reported increase in SAM patient keratinocyte cytokines was weighted toward a Th2 response (10, 14). However, the transcriptome analyses of both Dsg1-deficient animals and SAM biopsies revealed an inflammatory response that was skewed toward Th17 and upregulation of IL-36 response genes. Consistent with this observation, gene signatures from both the Dsg1-deficient animals and lesional skin from patients with SAM syndrome showed significant similarity to gene signatures from a cohort of patients with PSO and less so to a cohort of patients with AD. These findings have significant clinical implications; the treatment of 2 Dsg1-deficient patients with SAM syndrome with the IL-12/IL-23 antagonist ustekinumab resulted in marked improvement of skin lesions along with a recovery of the transcriptome to control levels. Interestingly, while morphological features of Dsg1-deficient epidermis resemble those found in human patients with pemphigus foliaceus (PF), the transcriptome of PF lesions showed weaker Th17 skewing than that resulting from targeting the *Dsg1* gene. As the IL-17/IL-23 skewed gene signature is present before birth in Dsg1-null embryos, genetically induced loss of Dsg1 could predispose individuals to skin inflammation.

## Results

### Dsg1^–/–^ mice exhibit loss of cell-cell adhesion and severe epidermal peeling in spite of increased Dsc1 and Dsg3.

Dsg1 stands out among the other desmosomal cadherins as having 3 genes instead of 1 in the mouse, *Dsg1a*, *b*, and *c*, located on chromosome 18 (24, 25). The functional differences among these 3 genes in various tissues are not well understood (25). qRT-PCR of *Dsg1^+/+^* mouse tissue revealed that only *Dsg1a* and *Dsg1b* genes are expressed in skin, as well as the esophagus, tongue, and forestomach, whereas *Dsg1c* expression was found only in the liver (Supplemental Figure 1A; supplemental material available online with this article; https://doi.org/10.1172/JCI144363DS1). To generate a Dsg1 knockout mouse, 2 different methods were employed using CRISPR/Cas9 technology (Supplemental Figure 1B). In one model, exon 2 was deleted in the *Dsg1a* and *Dsg1b* genes to mimic a previously characterized SAM syndrome mutation using gRNAs flanking exon 2 in each gene (10). Loss of exon 2, which was confirmed by PCR on genomic DNA, resulted in perinatal lethality of the chimeric animals. While we were unable to generate mice with germline transmission, blisters were observed in the skin of chimeric animals and histological analyses revealed areas of Dsg1 loss associated with epidermal blistering at the junction between the granular and cornified layers (Supplemental Figure 1, C and D). We also observed a reduction in total Dsg1 in the skin of chimeric animals by immunoblot, indicating that deletion of exon 2 causes reduced stability of Dsg1 (Supplemental Figure 1E).

**Figure 1:**
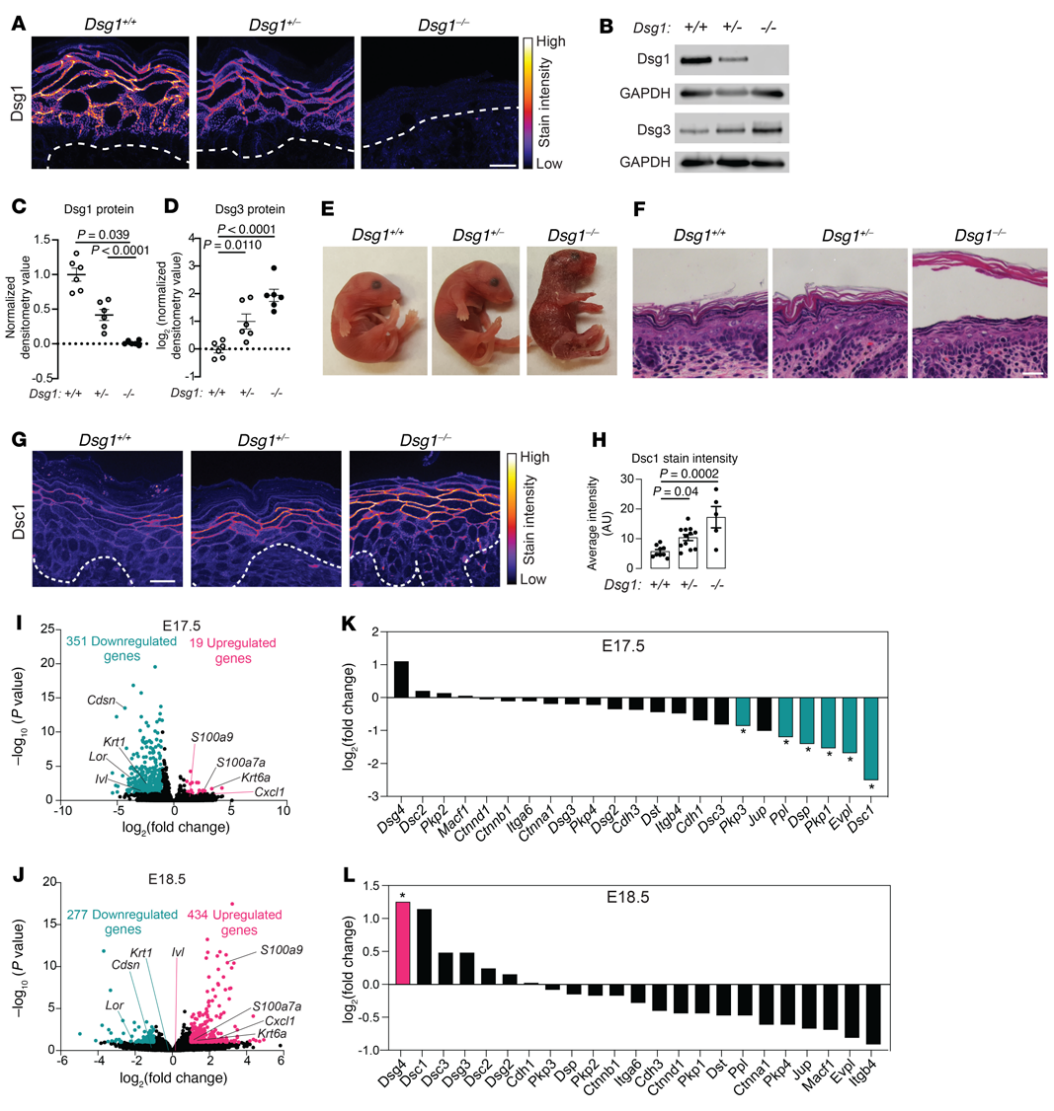
*Dsg1^–/–^* mice exhibit defects in epidermal adhesion and aberrant expression of adhesion proteins. (**A**) Immunostaining for Dsg1 in E18.5 mouse skin. Dashed line indicates location of the basement membrane. Scale bar = 20 μm (*n =* 3–5/genotype). (**B**) Immunoblot for Dsg1 and Dsg3 in protein extracts from E18.5 mouse skin. GAPDH was used as a loading control. (**C** and **D**) Quantification of Dsg1 (**C**) and Dsg3 (**D**) protein from immunoblots (data represent mean ± SEM, *n =* 6/genotype). Densitometry values were normalized to the *Dsg1^+/+^* samples and GAPDH. (**E**) Images of neonates shortly after birth (*n =* 3–7/genotype). (**F**) Histochemistry of skin from E18.5 mice. Scale bar = 20 μm (*n =* 20/genotype). (**G**) Immunostaining for Dsc1 in E18.5 skin. Dashed line represents basement membrane. Scale bar = 20 μm. (**H**) Staining intensity of Dsc1 in the epidermis in E18.5 mouse skin (data represent mean ± SEM, *n =* 5–12/genotype). (**I**) Volcano plot of upregulated and downregulated genes from RNA-Seq analysis performed on E17.5 skin. FDR ≤ 0.1 and log_2_ FC ≥ 1 considered significant (*n =* 4/genotype). (**J**) Volcano plot of upregulated and downregulated genes from RNA-Seq analysis performed on E18.5 skin (E18.5 data set #1, *n =* 4/genotype). FDR ≤ 0.1 and log_2_ FC ≥ 1 considered significant. (**K**) mRNA expression levels for proteins that make up desmosomes, adherens junctions, and hemidesmosomes from the E17.5 RNA-Seq data set (*FDR < 0.1). (**L**) mRNA expression levels for proteins that make up desmosomes, adherens junctions, and hemidesmosomes from the E18.5 time course RNA-Seq data set #1 (*FDR < 0.1). Statistical significance for **C**, **D**, and **H** was determined using 1-way ANOVA with a Tukey correction for multiple comparisons.

In the second model, the tandemly organized *Dsg1a*, *b*, and *c* gene cluster was removed by gene editing. Sequence analysis of progeny confirmed loss of the *Dsg1* gene cluster in knockout animals. A complete loss or reduction in Dsg1 protein (Figure 1, A–C) and mRNA was observed in *Dsg1*^–/–^ and *Dsg1^+/–^* animals, respectively (Supplemental Figure 2, A and B). *Dsg1^–/–^* animals are similar in size to their *Dsg1^+/+^* and *Dsg1^+/–^* littermates and are born at normal Mendelian ratios (151 *Dsg1^–/–^* animals/576 total animals analyzed). *Dsg1^–/–^* animals die within hours of birth and exhibit severe skin fragility and perinatal lethality with peeling skin, corresponding with the report of a similar Dsg1-deficient model (Figure 1E and ref. 22). Histologic analysis revealed a split between the granular and cornified layers of epidermis in 100% of the *Dsg1*^–/–^ animals (*n =* >20/genotype) with the stratum corneum (SC) separating completely from the tissue (Figure 1F). This phenotype was not observed in the *Dsg1^+/+^* or *Dsg1^+/–^* animals. Increased intercellular spaces were observed in the granular layer of *Dsg1^–/–^* animal epidermis by electron microscopy, suggesting that adhesion defects are also present in this layer (Supplemental Figure 2C).

Based upon the observed adhesion defects in the skin of *Dsg1^–/–^* animals, we determined levels of mRNA and protein for other desmosomal and classic cadherins and their associated proteins in the epidermis of E18.5 animals (Supplemental Figure 2, D and E). E-cadherin (Ecad, *Cdh1*), P-cadherin (Pcad, *Cdh3*), and desmoplakin (DP, *Dsp*) gene expression was unchanged and Ecad, DP, and plakoglobin (PG, *Jup*) protein levels and distribution within the epidermal layers were unchanged in *Dsg1^+/–^* and *Dsg1^–/–^* animals compared with *Dsg1^+/+^* animals. In contrast, the desmosomal cadherin Dsg3, which normally exhibits a reciprocal distribution pattern compared with Dsg1 (26), was increased at both total protein and gene expression levels in *Dsg1^+/–^* and *Dsg1*^–/–^ animals (Figure 1, B and D and Supplemental Figure 2, A, D, and E). Immunofluorescence staining showed an expanded Dsg3 distribution into the superficial spinous layers in *Dsg1*^–/–^ epidermis compared with the restricted distribution in basal layers of *Dsg1*^+/+^ epidermis (Supplemental Figure 2D). We also observed a corresponding redistribution of *Dsg3* mRNA in the *Dsg1^–/–^* epidermis by RNAscope (ACDBio), with *Dsg3* gene expression restricted to the basal layers in *Dsg1^+/+^* epidermis, while *Dsg3* expression expanded into the upper layers in *Dsg1^–/–^* epidermis (Supplemental Figure 2A). Staining intensity for Dsc1, which is normally expressed in the most suprabasal layers of the epidermis, was increased in *Dsg1*^–/–^ epidermis compared with *Dsg1^+/+^* (Figure 1, G and H), and *Dsc1* gene expression was increased in *Dsg1*^–/–^ skin (Supplemental Figure 2E).

**Figure 2:**
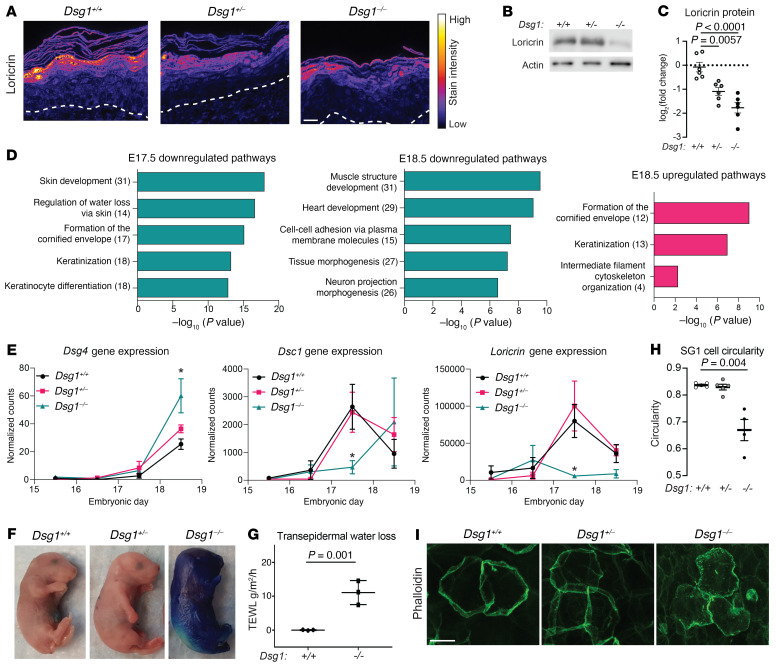
Dsg1 loss interferes with keratinocyte differentiation and epidermal barrier function. (**A**) Loricrin immunostaining in skin from E18.5 mice. Scale bar = 20 μm (*n =* 6–13/genotype). (**B**) Immunoblot for loricrin in protein extracts from E18.5 mouse skin. Actin was used as loading control. (**C**) Quantification of loricrin protein from immunoblot. Densitometry values were normalized to the *Dsg1^+/+^* samples and actin (data represent mean ± SEM, *n =* 6/genotype). (**D**) Gene Ontology (GO) Biological Process terms significantly overrepresented in downregulated genes in E17.5 and E18.5 *Dsg1^–/–^* skin and upregulated genes in E18.5 *Dsg1^–/–^* skin (E18.5 data set #1). Values in parentheses represent the number of genes associated with each pathway. (**E**) Normalized counts for barrier- forming genes *Dsg4*, *Dsc1*, and *Loricrin* from E15.5 to E18.5 RNA-Seq data sets (data represent mean ± SEM, *FDR < 0.1). (**F**) Toluidine blue barrier assays performed on E18.5 embryos demonstrating an outside-in barrier defect in *Dsg1^–/–^* animals (4–11/genotype). (**G**) Transepidermal water loss measured in P1 pups approximately 5 hours after birth (data represent mean ± SEM, 3/genotype). *P* value calculated using Student’s *t* test. (**H**) Quantification of cell circularity in the SG1 layer in epidermal whole mounts from E18.5 mice (data represent mean ± SEM, *n =* 4–5/genotype). (**I**) Representative images of phalloidin staining from epidermal whole mounts from E18.5 mice. Scale bar = 20 μm (*n =* 4–5/genotype). Statistical significance for **C** and **H** was determined using 1-way ANOVA with a Tukey correction for multiple comparisons.

**Figure 3:**
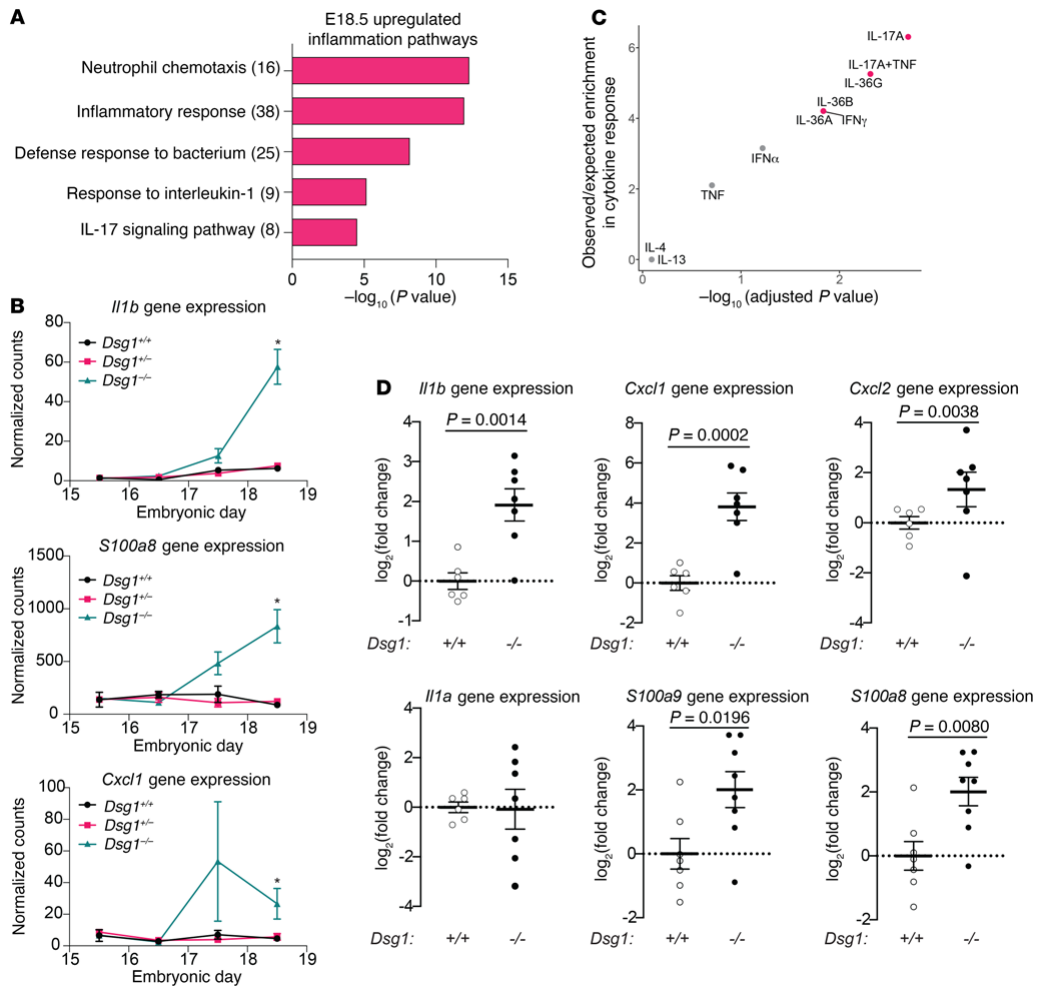
Dsg1 loss is associated with increased inflammatory response and antimicrobial pathways in E18.5 mouse epidermis. (**A**) GO Biological Process terms significantly overrepresented in upregulated genes involved in immune processes from E18.5 *Dsg1^–/–^* skin (data set #1). Values in parentheses represent the number of genes associated with each pathway. (**B**) Normalized counts of mRNA for selected inflammatory genes from E15.5 to E18.5 from the RNA-Seq data set #1 (data represent mean ± SEM, *FDR < 0.1). (**C**) Comparison between RNA-Seq data sets of cytokine-stimulated keratinocytes and *Dsg1^–/–^* E18.5 skin data set #1. Data are plotted as observed/expected ratio for enrichment in cytokine response as a function of the adjusted *P* value. Statistically significant similarities are indicated in red (adjusted *P* value < 0.05). (**D**) qRT-PCR for inflammatory cytokines and chemokines in mRNA from *Dsg1^+/+^* and *Dsg1^–/–^* E18.5 mouse skin. Fold change in gene expression was calculated using the ΔΔCT method, normalizing to GAPDH and then the *Dsg1^+/+^* mouse (data represent mean ± SEM, *n =* 6–8/genotype). *P* value calculated using Student’s *t* test.

To comprehensively interrogate gene expression differences between *Dsg1^+/+^,* *Dsg1^+/–^*, and *Dsg1^–/–^* animals, RNA-Seq analysis on RNA isolated from E15.5 to E18.5 skin was performed to analyze changes in transcriptional profiles during development (data set #1). Comparisons include another independent round of RNA-Seq performed at E18.5 (data set #2). Few changes in gene expression were observed between the *Dsg1^+/+^* animals and the *Dsg1^–/–^* animals at E15.5 and E16.5 (Supplemental Figure 3, A and B). We observed 19 significantly upregulated and 351 significantly downregulated genes in *Dsg1^–/–^* mice compared with *Dsg1^+/+^* mice at E17.5 (FDR ≤ 0.1 and log_2_ fold change (FC) ≥ 1), whereas 434 significantly upregulated and 277 significantly downregulated genes were observed in *Dsg1^–/–^* animals compared with *Dsg1^+/+^* in E18.5 mice (Figure 1, I and J). An independent data set obtained at E18.5 had 107 significantly upregulated and 91 significantly downregulated genes in *Dsg1^–/–^* animals compared with *Dsg1^+/+^* (Supplemental Figure 3C). There was substantial overlap in the gene signatures exhibited by E18.5 data sets #1 and #2 (Supplemental Figure 3D; also compare Supplemental Figure 3, E–G with Figure 1L, Figure 2D, Figure 3A, and Supplemental Figure 4A). For comparative analyses, both E18.5 data sets were considered as detailed below.

As there were few changed genes in *Dsg1^+/–^* animals compared with *Dsg1^+/+^* animals at E17.5 and E18.5, all further analyses of the RNA-Seq data set were performed using the *Dsg1^–/–^* animals. This observation of limited changes in *Dsg1^+/–^* animals is consistent with no observable gross phenotype at baseline in these animals. Analysis of genes involved in forming desmosomes, adherens junctions, and hemidesmosomes revealed a significant decrease in expression of mRNAs encoding for several desmosomal proteins, including *Dsc1*, *Pkp1*, *Dsp*, and *Pkp3* at E17.5 (Figure 1K). Analysis of these same genes at E18.5 in both RNA-Seq data sets revealed that many of the desmosomal genes returned to normal expression levels. *Dsg4* was significantly increased in expression in both E18.5 data sets and *Dsc1* was significantly increased in data set #2 and modestly upregulated in data set #1 (Figure 1L and Supplemental Figure 3G). *Dsg3* and *Dsg2* were also significantly increased in data set #2 (Supplemental Figure 3G). While there were some differences observed between the RNA-Seq versus qRT-PCR data for significantly changed genes at E18.5, genes trended in the same direction across both methods (Figure 1L, Supplemental Figure 2E, and Supplemental Figure 3G).

### The epidermal differentiation program and barrier function are disrupted in Dsg1^–/–^ animals.

The severe epidermal peeling and observed disruption of epidermal adhesion, along with our previous work showing Dsg1 promotes keratinocyte differentiation in vitro (19), prompted us to assess differentiation and barrier functions in the *Dsg1^–/–^* animals. Loricrin, a structural component of the cornified envelope, is decreased in E18.5 *Dsg1^–/–^* mice as observed by immunoblot and immunofluorescence, without a change in mRNA levels (Figure 2, A–C and Supplemental Figure 4E). Interestingly, not all differentiation-associated proteins are affected at E18.5, as protein levels of involucrin and transglutaminase, while variable, are unchanged in the epidermis of *Dsg1^–/–^* mice even though they are increased at gene expression in *Dsg1^–/–^* mice (Supplemental Figure 4, B–E).

To address on a more global scale whether Dsg1-deficient mice exhibit abnormalities in epidermal differentiation and barrier function, we carried out further analysis of the RNA-Seq data from E17.5 and E18.5 mice. Functional enrichment analysis of downregulated genes in E17.5 mice revealed pathways involved in skin development, keratinization, and keratinocyte differentiation (Figure 2D). These pathways were no longer enriched in the downregulated genes in E18.5 mice, while pathways involved with cell-cell adhesion via plasma membrane molecules and tissue morphogenesis were observed (Figure 2D). Functional enrichment analysis of the upregulated genes in E18.5 *Dsg1^–/–^* data set #1 revealed pathways involved with formation of the cornified envelope, keratinization, and intermediate filament cytoskeleton organization (Figure 2D). These pathways were also associated with the upregulated genes from the E18.5 data set #2 (Supplemental Figure 4A). Analysis of individual genes regulated upon keratinocyte differentiation from E15.5 to E18.5, including *Dsg4*, *Dsc1*, and *Loricrin*, reveal distinct patterns of expression in *Dsg1^–/–^* mice. *Dsg4* is unchanged until E18.5, where its gene expression is significantly increased (Figure 2E). While *Dsc1* is significantly decreased at E17.5, it shows a trend toward increased expression at E18.5. *Loricrin* is significantly decreased in gene expression at E17.5 but unchanged at E18.5. These observations suggest that there is a delay in the transcriptional differentiation program at E17.5, which either returns to normal or is increased at E18.5. Changes in genes involved in keratinocyte differentiation were validated by qRT-PCR (Supplemental Figure 4E).

To complement the RNA-Seq and protein analysis indicating a potential barrier defect in *Dsg1^–/–^* animals, we carried out barrier function assays. An increase in toluidine blue dye penetration, a measure of the outside-in barrier, was observed in E18.5 *Dsg1^–/–^* animals (Figure 2F). We also observed an increase in transepidermal water loss, a measure of inside-out barrier, in P1 *Dsg1^–/–^* animals (Figure 2G), corresponding with changes in tight junctions in both mouse and human models of Dsg1 deficiency (22, 27). We next analyzed changes in cell shape in the stratum granulosum 1 (SG1) layer in the epidermis at E18.5 by staining skin whole mounts for F-actin, which labels cells in the SG1 layer brightly. Circularity of cells in the SG1 layer was significantly reduced in *Dsg1^–/–^* animals compared with *Dsg1^+/+^* animals (Figure 2, H and I). This observation is consistent with our previous findings of changes in cell shape in Dsg1-deficient 3D epidermal cultures and suggests irregular packing of cells in this layer (19).

### E18.5 Dsg1^–/–^ mouse and human SAM syndrome skin share Th17 skewed inflammatory signatures.

In addition to genes involved in skin differentiation and barrier function, pathway analysis of upregulated genes in E18.5 *Dsg1^–/–^* animals revealed genes involved in inflammatory processes including neutrophil chemotaxis, defense response to bacterium, response to IL-1, and IL-17 signaling pathway (Figure 3A). Analysis of a subset of genes involved in these inflammatory pathways (*Il1b*, *S100a8*, and *Cxcl1*) exhibited a modest but nonsignificant increase in expression at E17.5, when differentiation and adhesion-related genes were depressed compared with *Dsg1^+/+^* mice, with a significant increase in expression at E18.5 (Figure 3B). The E18.5 *Dsg1^–/–^* transcriptome was compared with the transcriptome from individual keratinocyte cultures that were treated with recombinant human cytokines as described (28). The E18.5 *Dsg1^–/–^* animal RNA-Seq (data set #1) exhibited significant similarities to keratinocytes stimulated with IL-17A, IL17A + TNF, IL-36A, and IL-36G, while there was no overlap with keratinocytes treated with IL-13 or IL-4 (Figure 3C). Genes present in these pathways were validated by qRT-PCR for *Il1a* *Il1b*, *Cxcl1*, *Cxcl2*, *S100a8*, and *S100a9* (Figure 3D). The fact that this analysis was performed on embryonic skin raises the possibility that loss of Dsg1, in the absence of an external stimulus, primes a proinflammatory program in keratinocytes.

**Figure 4:**
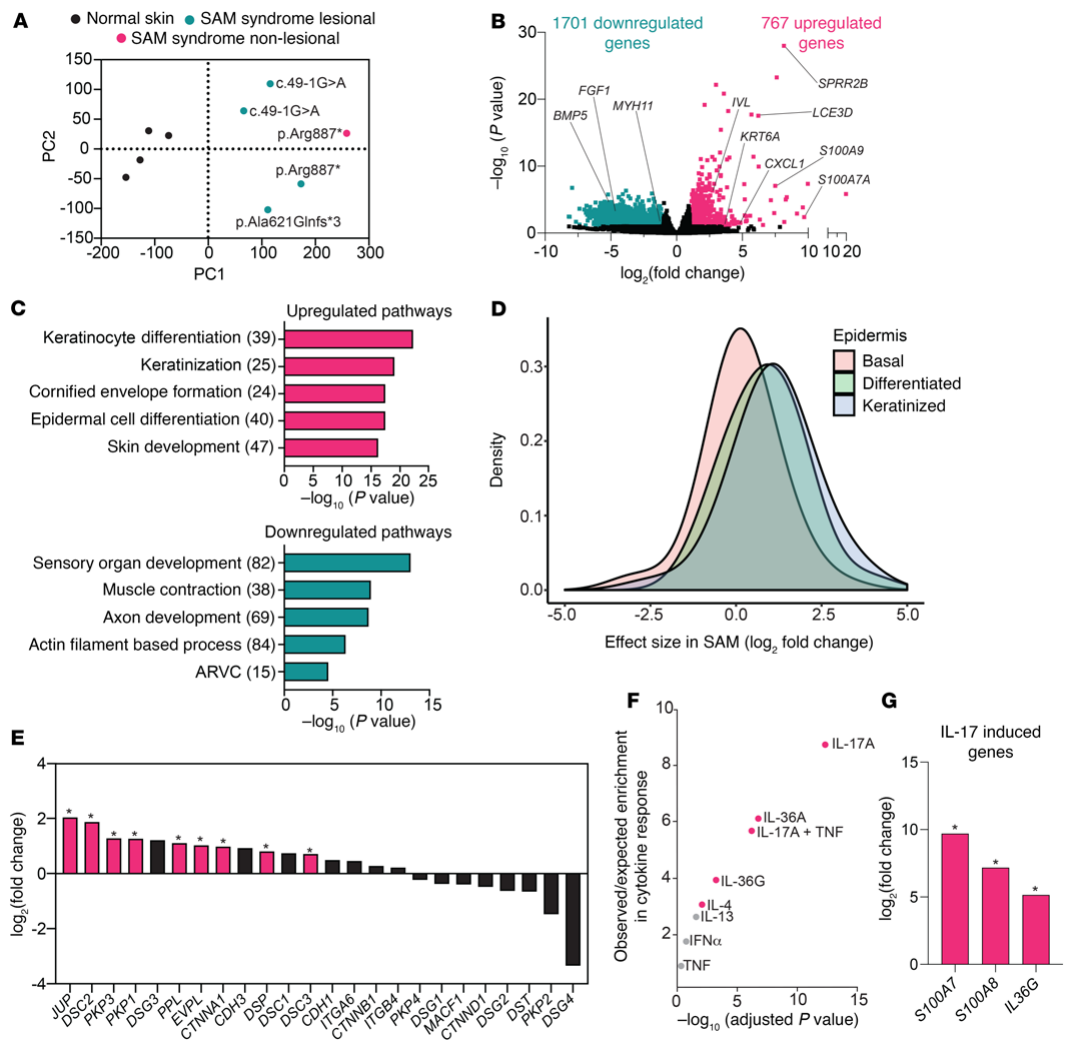
SAM syndrome patient skin whole transcriptome profile shows similarities to that of the *Dsg1^–/–^* mouse. (**A**) PCA for SAM syndrome patient samples: 4 from lesional skin, 1 from nonlesional skin, and 4 normal skin whole transcriptome profiles. (**B**) Volcano plot of upregulated (767) and downregulated (1701) genes in RNA-Seq data from SAM lesional skin biopsy sections. FDR ≤ 0.1 and log_2_ FC ≥ 1 was considered significant. (**C**) GO Biological Process terms significantly overrepresented in upregulated or downregulated genes from patients with SAM syndrome. Values in parentheses represent the number of genes associated with each pathway. (**D**) SAM syndrome RNA-Seq data were compared with a single-cell RNA-Seq data set from skin. The level of expression for each gene was determined in normal keratinocytes at different stages of differentiation and the expression level of these genes in SAM syndrome was graphed. (**E**) Gene expression for desmosomal, adherens junction, and hemidesmosome genes in the SAM data set (*FDR < 0.1). (**F**) Upregulated gene signatures from SAM syndrome RNA-Seq compared with those from keratinocytes that had been treated with cytokines in culture. Statistically significant similarities are indicated in red (adjusted *P* value < 0.05). (**G**) Gene expression for IL-17–induced genes in the SAM syndrome data set (*FDR < 0.1).

To address the role of Dsg1 in humans, we performed whole transcriptome analysis on lesional skin biopsies taken from 4 patients with SAM syndrome with 3 different *Dsg1* mutations that result in the improper expression and/or delivery of the protein to the cell-cell membrane. These mutations include a nonsense mutation causing a premature stop codon, truncating part of the cytoplasmic domain of Dsg1 at amino acid 887, a frame shift mutation, resulting in a truncation of the Dsg1 cytoplasmic domain at amino acid 621, and a mutation leading to exon 2 skipping, which removes the Dsg1 signal sequence and leads to Dsg1 mislocalization (Supplemental Figure 5 and refs. 10, 12, 29). Principle component analysis (PCA) revealed that the lesional samples from the 4 patients with SAM syndrome cluster away from the 4 control samples, with the 2 c.49-1G>A mutant sibling samples clustered together (Figure 4A). Whether this clustering is due to genetic similarities or biopsy site is not known. However, most SAM syndrome samples were taken from the same body site, posterior thigh, except the p.Arg887* patient sample, which was taken from the lower back (30). We also performed RNA-Seq on a nonlesional sample paired with a lesional sample from 1 patient. PCA showed that this nonlesional sample was separated from normal control samples and lesional samples. We observed 767 upregulated and 1701 downregulated genes (FDR ≤ 0.1 and log_2_ FC ≥ 1) in lesional skin compared with control samples (Figure 4B).

Like what was observed in the *Dsg1^–/–^* mouse skin, gene ontology (GO) pathway analysis on upregulated genes from patients with SAM syndrome revealed pathways involved in skin development, differentiation, keratinization, and cornified envelope formation (Figure 4C). Downregulated genes from patients with SAM syndrome were enriched for pathways involving neuron development, including sensory organ development and axon development, as well as pathways associated with the actin cytoskeleton (Figure 4C). Comparison to a single-cell RNA-Seq data set from skin revealed that genes associated with differentiated and keratinized keratinocytes are upregulated in the skin in patients with SAM syndrome, potentially due to an increase in the representation of these cells or a general upregulation of these genes in the upper epidermal layers (Figure 4D). Like the *Dsg1^–/–^* animals, patients with SAM syndrome exhibited alterations in desmosomal associated proteins. In both, desmosomal cadherins were frequently increased (Figure 1, K and L, Supplemental Figure 3G, and Figure 4E). An exception is *DSG4,* which was decreased in SAM syndrome and increased significantly in *Dsg1^–/–^* animals. One plausible explanation for this is that a p63 enhancer upstream of the 3 tandem *Dsg1* genes is brought into close proximity to the *Dsg4* gene in *Dsg1^–/–^* mice, possibly impacting its expression (31). In contrast, several plaque proteins that were significantly increased in SAM syndrome trended downward in E18.5 animals, including *JUP*, *PKP1*, *PPL*, and *DSP* (Figure 4E). While we do not have an explanation for these differences, it seems plausible that external mechanical stress experienced by human skin that occurs to a lesser extent during mouse embryogenesis could have an impact on plaque protein expression.

Comparison of upregulated genes in SAM syndrome with genes upregulated in cytokine-stimulated keratinocytes revealed a significant similarity to IL-17A responses, like what was observed in mouse *Dsg1^–/–^* skin (Figure 4F and Figure 3C). While IL-17A signaling has been implicated in both allergic inflammation and PSO, there was less enrichment of genes involved in the proallergic IL-4 and IL-13 responses, despite the fact that patients with SAM syndrome often develop food allergies and have elevated levels of IgE (10, 23). Keratinocyte genes that are upregulated by IL-17 and are also upregulated in patients with SAM syndrome include *S100A8*, *S100A7*, and *IL36G* (Figure 4G).

### PF patient skin exhibits a weaker inflammatory profile than that shared by Dsg1^–/–^ mice and patients with SAM syndrome.

**Figure 5:**
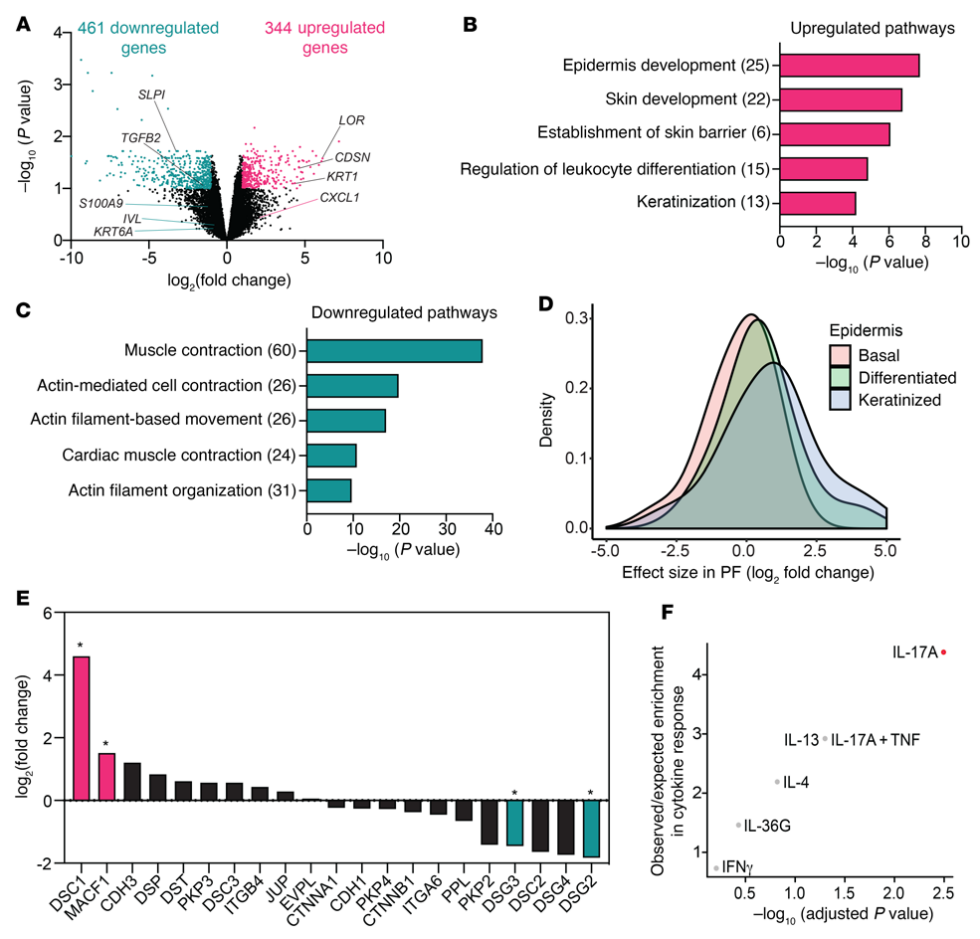
Whole transcriptional analysis of skin from patients with PF reveals profiles that are distinct from that of genetic Dsg1 deficiency. (**A**) Volcano plot of upregulated (344) and downregulated (461) genes in RNA-Seq data from PF patient samples. FDR ≤ 0.1 and log_2_ FC ≥ 1 was considered significant. (**B** and **C**) GO Biological Process terms significantly overrepresented in upregulated (**B**) and downregulated (**C**) genes from patients with PF. Values in the parentheses represent the number of genes associated with each pathway. (**D**) PF patient RNA-Seq was compared with a single-cell RNA-Seq data set from skin. The level of expression for each gene was determined in normal keratinocytes at different stages of differentiation and the expression level of these genes in PF was graphed. (**E**) Gene expression for desmosomal, adherens junction, and hemidesmosome genes in the PF data set (*FDR < 0.1). (**F**) Upregulated gene signatures from PF RNA-Seq compared with those from keratinocytes that had been treated with cytokines in culture. Statistically significant similarities are indicated in red (adjusted *P* value < 0.05).

PF is an autoimmune disorder in which circulating anti-Dsg1 autoantibodies bind to Dsg1 and interfere with cell-cell adhesion through steric hindrance and/or by stimulating endocytic turnover (6). Autoantibody-mediated interference with Dsg1 results in loss of epidermal adhesion, which appears similar to that caused by genetic loss of Dsg1. Therefore, PF offered an opportunity to address whether both genetic and autoantibody-induced interference with Dsg1 adhesion result in similar transcriptome remodeling. While transcriptome and cytokine analyses of circulating lymphocytes isolated from patients with PF have been reported, the transcriptome of lesional skin from patients with PF has not been investigated (32). Therefore, we carried out RNA-Seq on samples from 7 patients with PF. Compared with control samples, there were 461 significantly downregulated and 344 significantly upregulated genes in PF patient samples (FDR ≤ 0.1 and log_2_ FC ≥ 1, Figure 5A). Pathways revealed by GO analysis of significantly upregulated genes included epidermis development, establishment of skin barrier, and keratinization (Figure 5B). Immune response pathways, such as regulation of leukocyte differentiation, were also observed in PF patient samples (Figure 5B). However, these pathways differed from those seen in SAM syndrome or the *Dsg1^–/–^* mouse (Figure 4C and Figure 3A). Pathway analysis of downregulated genes revealed an enrichment for actin- and muscle-related processes and actin filament–based movement (Figure 5C). Comparison to a single-cell RNA-Seq data set from skin revealed genes expressed by keratinized keratinocytes were generally upregulated in PF skin, without an enrichment in genes representing other keratinocyte populations (Figure 5D). The increase in keratinized genes (late differentiation) is similar to that observed in SAM syndrome. However, SAM syndrome also had an increase in keratinocyte-specific genes, representing earlier differentiation stages, that was not observed in PF (Figure 4D). Analysis of cadherin and cadherin-associated genes in PF patient samples revealed similarities to the *Dsg1^–/–^* mouse, including a significant increase in *DSC1* gene expression, with differences including significant downregulation of *DSG3* in patients with PF compared with its upregulation in *Dsg1^–/–^* skin (Figure 5E, Supplemental Figure 2, A and E, and Supplemental Figure 3G). Comparison of the PF gene signature to cytokine-stimulated keratinocyte gene signatures revealed a moderate but significant overlap with IL-17A–treated cells. However, the strength of the association was more modest in PF than in SAM syndrome or the *Dsg1^–/–^* mouse (Figure 5F). In contrast to SAM syndrome and the *Dsg1^–/–^* mouse, no association with IL-36G or IL-36A responses was observed in PF patient samples.

**Figure 6:**
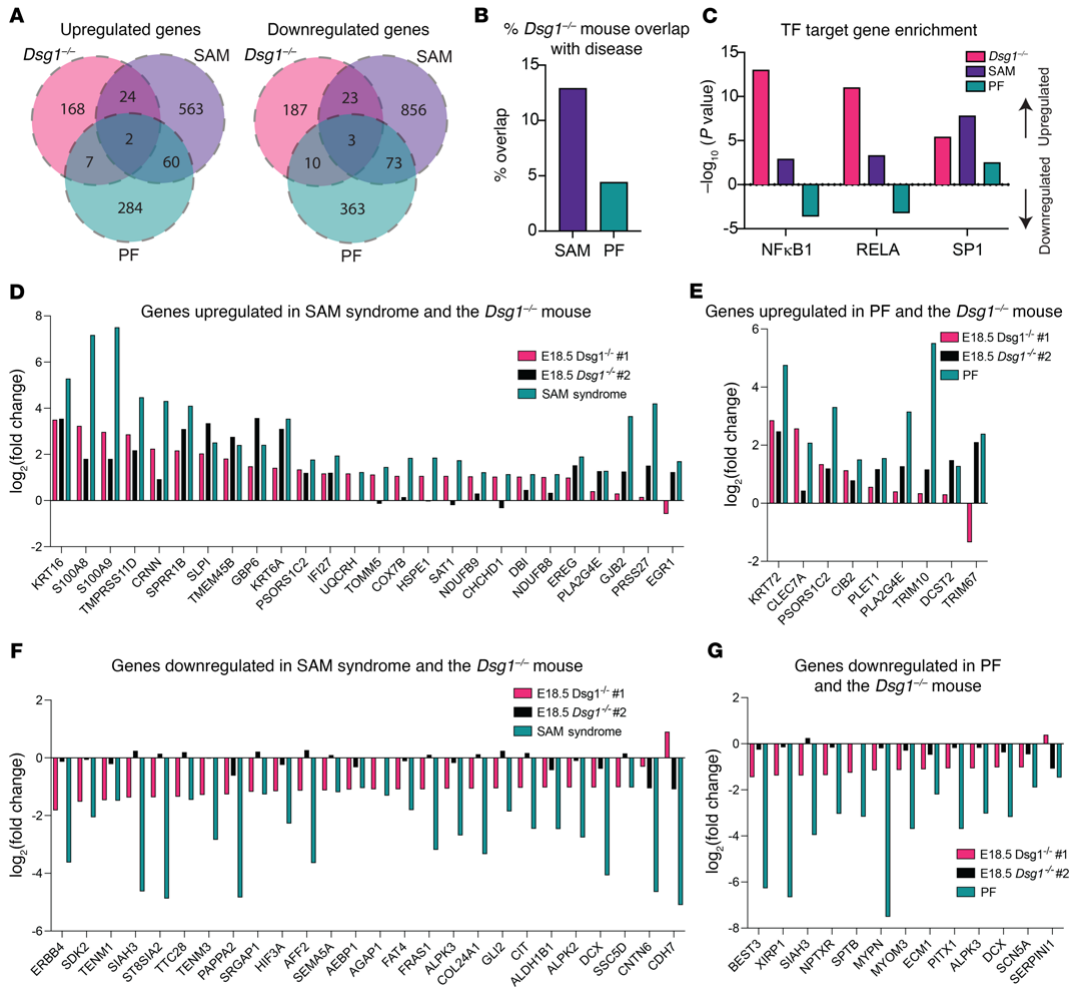
Differentially expressed genes from *Dsg1^–/–^* mice are shared with patients with SAM syndrome to a greater degree than with patients with PF. (**A**) Overlap between genes upregulated or downregulated in the *Dsg1^–/–^* mouse skin (E18.5 data sets #1 and #2), and patients with SAM syndrome or PF. (**B**) Percentage of genes upregulated in the *Dsg1^–/–^* mouse data sets #1 and #2 and patients with SAM syndrome or PF. (**C**) Predicted transcription factor activity of NFκB1, RELA, and SP1. *P* values above the *x* axis represent enrichment of genes targeted by the transcription factor in the upregulated genes, while *P* values below the *x* axis represent enrichment of transcription factor targets in the downregulated genes (E18.5 data sets #1 and #2, *P* < 0.05 considered significant). (**D**) Overlap between genes upregulated in *Dsg1^–/–^* mouse skin (E18.5 data sets #1 and #2) and SAM syndrome. (**E**) Overlap between genes upregulated in *Dsg1^–/–^* mouse skin (E18.5 data sets #1 and #2) and PF. (**F**) Overlap between genes downregulated in *Dsg1^–/–^* mouse skin (E18.5 data sets #1 and #2) and SAM syndrome. (**G**) Overlap between genes downregulated in *Dsg1^–/–^* mouse skin (E18.5 data sets #1 and #2) and PF.

Additional comparisons between the *Dsg1^–/–^* animals and SAM syndrome or PF revealed that 12.9% of the genes significantly upregulated in *Dsg1^–/–^* skin were also significantly upregulated in lesional skin from patients with SAM syndrome, and 11.7% of significantly downregulated genes in the *Dsg1^–/–^* skin were also significantly downregulated in patients with SAM syndrome (Figure 6, A, B, D, and F). Genes significantly upregulated in both SAM syndrome and *Dsg1^–/–^* skin included genes involved in antimicrobial responses in the skin (*S100A8*, *S100A9*, and the secretory leukocyte proteinase inhibitor of neutrophil elastase [*SLPI*]) (Figure 6D). The overlap in expression patterns was present even though comparisons were between human skin from adults or adolescents and embryonic mouse skin, suggesting that Dsg1 regulates inflammatory pathways independently of an external stimulus, priming the skin for a proinflammatory response. Comparison between *Dsg1^–/–^* skin and PF showed that 4.4% of genes significantly upregulated in the mouse were also significantly upregulated in PF, and 5.8% of genes significantly downregulated in the Dsg1 mouse were also downregulated in PF (Figure 6, A, B, E, and G). None of the inflammatory genes upregulated in the *Dsg1^–/–^* mouse and in patients with SAM syndrome, such as *S100A8*, *S100A9*, and *SLPI*, were upregulated in PF patient skin. Thus, while all conditions are associated with changes in keratinocyte differentiation pathways, SAM syndrome and the *Dsg1^–/–^* mouse share greater similarities in inflammatory signatures compared with PF.

We also analyzed predicted transcription factor targets on the upregulated and downregulated genes from all 3 data sets using the TRRUST database (33). Among several predicted transcription factors, we observed an association with target genes of the NFκB family transcription factors NFκB1 and RELA in upregulated genes in both *Dsg1^–/–^* mice and SAM syndrome (Figure 6C). We previously showed that Dsg1 can inhibit NFκB activity induced by TNF and IL-1β, supporting these observations that a loss of Dsg1 may increase NFκB activity (14). We also observed that NFκB1 and RELA target genes were associated with the downregulated genes in patients with PF (Figure 6C), suggesting that targeting Dsg1 by autoantibodies does not block its ability to downregulate NFκB activity, unlike genetic loss such as that observed in the *Dsg1^–/–^* mouse and SAM syndrome. Activity of the transcription factor SP1 was also associated with upregulated genes in all 3 Dsg1-targeted disorders, consistent with its roles in epidermal differentiation (34).

### Inflammatory profiles in Dsg1^–/–^ skin and SAM syndrome are more similar to PSO than AD.

**Figure 7:**
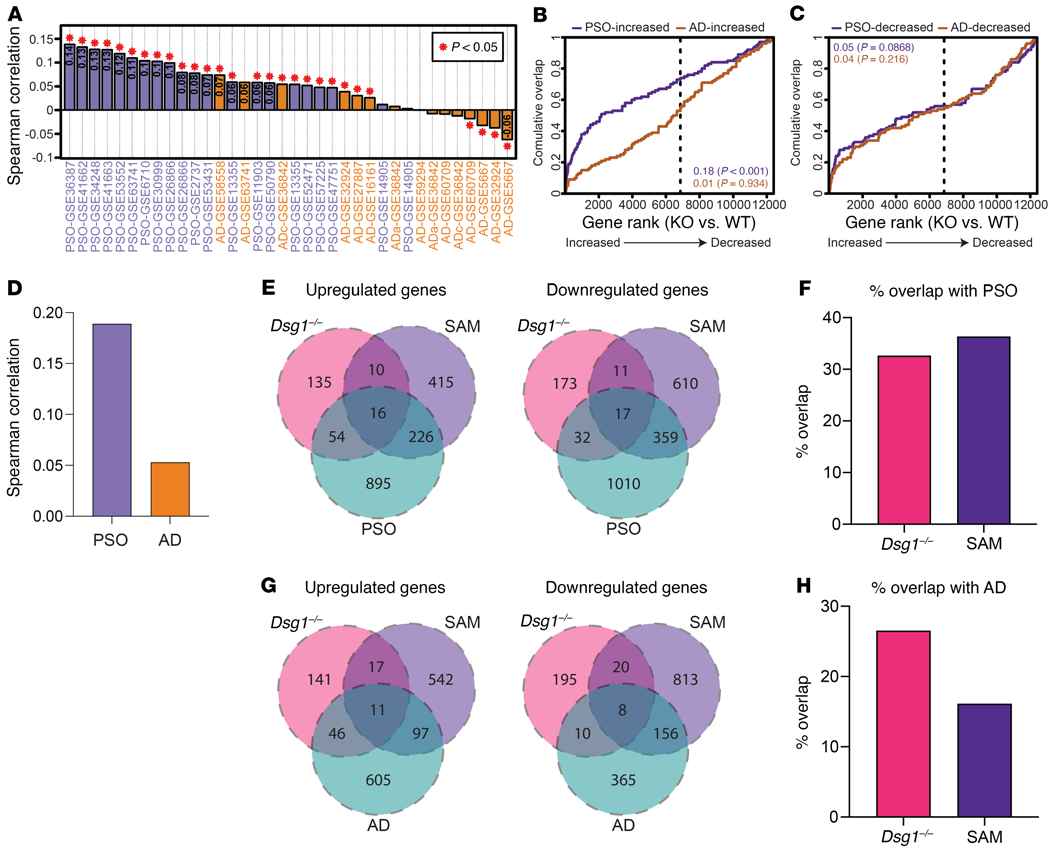
Differentially expressed genes in *Dsg1^–/–^* mice and patients with SAM syndrome overlap with patients with PSO and to a lesser extent those with AD. (**A**) The gene fold change signature (*Dsg1^–/–^*/*Dsg1^+/+^*) from E18.5 data set #2 was compared with those obtained from 36 comparisons yielding PSO/control (*n =* 21) or AD/control (*n =* 15) gene fold change signatures (ADa = acute atopic dermatitis; ADc = chronic atopic dermatitis). The 36 comparisons are ranked based upon the Spearman correlation coefficient estimate. (**B**) GSEA analysis of PSO/AD-increased genes. (**C**) GSEA analysis of PSO/AD-decreased genes. In **B** and **C**, the top 100 genes most strongly increased or decreased in each disease were analyzed. The figure shows cumulative overlap of these genes (vertical axis) with genes ranked based on *Dsg1^–/–^*/*Dsg1^+/+^* FC estimates (horizontal axis). The area between each curve and the diagonal is shown with corresponding *P* values (Wilcoxon rank sum test). (**D**) Gene fold change signatures (*Dsg1^–/–^*/*Dsg1^+/+^*) from E18.5 data set #1 were compared with the PSO and AD gene fold change signatures and the Spearman correlation was graphed to demonstrate correlation. (**E**) Overlap between genes upregulated or downregulated in the *Dsg1^–/–^* mouse skin and patients with SAM syndrome or PSO. (**F**) Percentage overlap of genes upregulated in E18.5 *Dsg1^–/–^* mouse skin and patients with SAM syndrome or PSO. (**G**) Overlap between genes upregulated or downregulated in the E18.5 *Dsg1^–/–^* mouse skin and patients with SAM syndrome or AD. (**H**) Percentage overlap of genes upregulated in E18.5 *Dsg1^–/–^* mouse skin and patients with SAM syndrome or AD.

Since inflammatory signatures exhibited by *Dsg1^–/–^* animals and patients with SAM syndrome are reminiscent of those in common inflammatory disorders such as PSO and AD, we compared transcriptional profiles from the *Dsg1^–/–^* animals and SAM syndrome with those from patients with PSO or AD. As the inflammatory signatures in PF were not as strong as those observed in the *Dsg1^–/–^* animals or SAM syndrome, we focused on the signatures from the *Dsg1^–/–^* animals and patients with SAM syndrome. First, we compared the E18.5 *Dsg1^–/–^* transcriptome from data set #2 to those from 36 patients with PSO or AD. After pairing mouse genes with human orthologs, the top 12 patients with the strongest resemblance to *Dsg1^–/–^* mice were from PSO comparisons (Figure 7A). Conversely, the 8 bottom-ranked with weakest resemblance (rs ≤ –0.00011) were AD comparisons. Consistent with this, the 100 genes most strongly elevated in PSO lesions overlapped significantly with those geneselevated in *Dsg1^–/–^* animals. This was not the case for genes elevated in AD lesions (Figure 7B). In contrast, the 100 genes decreased in PSO or AD lesions did not overlap significantly with those genes altered in *Dsg1^–/–^* mice (Figure 7C). We validated this observation by comparing the E18.5 *Dsg1^–/–^* transcriptome from data set #1 with the PSO and AD data sets and found a greater correlation with the PSO data set than with the AD data set (Figure 7D and Supplemental Figure 6, A and B). Direct comparison of the combined E18.5 *Dsg1^–/–^* skin data sets with PSO shows 32.6% of genes upregulated in *Dsg1^–/–^* skin are also upregulated in PSO patient samples and 21% of genes downregulated in the *Dsg1^–/–^* skin are also downregulated in patients with PSO (Figure 7, E and F). Genes upregulated in both PSO patient samples and *Dsg1^–/–^* skin are involved with antimicrobial response (e.g., *S100A8,* *S100A9*, *SLPI*, *LCN2*) and leukocyte chemotaxis (e.g., *CXCL2*, *CXCR2*; Supplemental Tables 1 and 2). Comparison of whole transcriptome data from patients with SAM syndrome with patients with PSO showed a correlation between differentially expressed genes (Figure 7, E and F). Direct comparison of significantly changed genes found that 36.3% of genes upregulated in SAM syndrome are also upregulated in PSO, and 37.7% of genes downregulated in SAM syndrome are downregulated in PSO (Figure 7, E and F). When compared with cytokine response gene sets, we observed a significant enrichment in IL-17A, IL-36A, and IL-36G response genes in patients with PSO, with no enrichment of IL-4 or IL-13 response genes (Supplemental Figure 6C), as observed in SAM syndrome and the *Dsg1^–/–^* mouse (Figure 3C and Figure 4F).

To further explore the overlap between the *Dsg1^–/–^* mouse, SAM syndrome, and PSO, we performed functional enrichment analysis on sets of genes that were overlapping between PSO and the *Dsg1^–/–^* mouse or PSO and SAM syndrome. Functional enrichment analysis on genes that were upregulated both in the *Dsg1^–/–^* mouse and in PSO revealed pathways including leukocyte migration, neutrophil degranulation, and IL-17 signaling (Supplemental Figure 6D and Supplemental Tables 1 and 2). Functional enrichment analysis on upregulated genes shared by PSO and SAM syndrome revealed pathways including cell cycle, neutrophil degranulation, response to bacterium, and keratinization (Supplemental Figure 6E). These observations show that genes involved in inflammatory pathways upregulated in SAM syndrome and the *Dsg1^–/–^* mouse are also upregulated in PSO. Interestingly, the percentage of differentially upregulated genes shared by the *Dsg1^–/–^* mouse and SAM was lower than the percentage shared by the *Dsg1^–/–^* mouse and PSO, 12.9% versus 32.6%, respectively (Figure 7, E and F and Supplemental Tables 1 and 2). To further explore the underlying basis for this difference in overlap, we performed functional enrichment analysis on differentially expressed genes shared by SAM and PSO but not *Dsg1^–/–^*, and differentially expressed genes shared by *Dsg1^–/–^* and PSO but not SAM. Pathways associated with inflammation, including neutrophil degranulation, and regulation of defense response were found in both sets of genes, highlighting the similarities in gene signatures across the mouse and human deficiencies even though the genes were different (Supplemental Figure 6, F and G and Supplemental Tables 1–4). We also observed some unique pathways, particularly in the SAM syndrome data set, including cell-cycle and epidermis development. Cell-cycle pathways may be present in SAM syndrome and PSO due to epidermal thickening, which we do not observe in the *Dsg1^–/–^* animals (Supplemental Figure 6, E and G).

Direct comparison of the E18.5 *Dsg1^–/–^* mouse with AD revealed 26.5% of genes upregulated in the *Dsg1^–/–^* mouse are also upregulated in AD, whereas only 7.7% of genes downregulated in the *Dsg1^–/–^* mouse are also downregulated in AD (Figure 7, G and H and Supplemental Tables 5–8). Functional enrichment analysis of genes upregulated in the *Dsg1^–/–^* mouse and AD identified both inflammatory pathways, such as leukocyte migration and response to bacterium, and keratinocyte differentiation pathways such as formation of the cornified envelope and keratinization (Supplemental Figure 6H and Supplemental Tables 5 and 6). Direct comparison of SAM syndrome with AD revealed 16.2% of genes upregulated in SAM syndrome are also upregulated in AD, whereas 16.5% of genes downregulated in SAM syndrome are also downregulated in AD (Figure 7, G and H and Supplemental Tables 5 and 7). Functional enrichment analysis of genes upregulated in both SAM syndrome and AD reveal pathways associated with keratinocyte differentiation, such as keratinization, formation of the cornified envelope, and immune pathways such as response to bacterium (Supplemental Figure 6I). Like what was observed with PSO, there was an enrichment in inflammatory pathway genes shared by AD and SAM or the *Dsg1^–/–^* mouse, however the strength of enrichment was lower than what was observed in genes overlapping with PSO (Supplemental Figure 6, H and I and Supplemental Tables 5 and 6). To further explore the overlap in inflammatory pathways we compared the keratinocyte cytokine response gene sets with AD and found an enrichment of IL-17A, IL-36A, IL-36G, IL-13, and IL-4 responses (Supplemental Figure 6J). These observations show that AD has enrichment in IL-17 response genes, like PSO, SAM syndrome, and the *Dsg1^–/–^* mouse, but AD uniquely has enrichment for IL-13 and IL-4 response genes. Genes associated with keratinization were found to be regulated similarly across the *Dsg1^–/–^* mouse, SAM syndrome, AD, and PSO (Supplemental Figure 6K). Taken together, these observations demonstrate that the inflammatory profile in SAM syndrome and the *Dsg1^–/–^* mouse is more similar to PSO, with an immune response dominated by IL-17, IL-36G, and IL-36A. While AD shares these cytokine responses it also contains IL-4 and IL-13 responses. Changes in pathways associated with keratinocyte differentiation however are generally shared across all conditions.

### Loss of Dsg1 is associated with an increase in S100A9 in tissue.

To validate our observations of shared inflammatory profiles between SAM syndrome, the *Dsg1^–/–^* mouse, and PSO, we stained skin samples from patients with SAM syndrome, PF, or PSO for S100A9. S100A9 is an antimicrobial peptide known to be elevated in PSO (35). *S100A9* mRNA is also increased in SAM syndrome and the *Dsg1^–/–^* mouse (Figure 6D). While inflammatory signatures were more modest in PF we also tested if S100A9 levels were increased to distinguish whether genetic or antibody-mediated loss of Dsg1 had differential effects on S100A9 levels. As previously described, S100A9 protein was increased in the skin of patients with PSO compared with control, with high intensity nuclear staining present in the full thickness of the epidermis (Supplemental Figure 7, A and B and ref. 35). High intensity nuclear S100A9 staining was also observed in SAM syndrome patient skin, but less so in PF patient skin. Percentage of S100A9-positive nuclei was increased in PSO and SAM syndrome compared with control (Supplemental Figure 7B). We also observed an increase in S100A9 protein by immunoblot in the E18.5 *Dsg1^–/–^* mouse skin (Supplemental Figure 7C). Like what was observed with the cytokine response pathway enrichment, PF samples trended toward an increase in nuclear S100A9, suggesting that PF does have a weaker inflammatory response in the skin, which shares features with SAM syndrome and PSO (Supplemental Figure 7, A and B).

### Dsg1 downregulation is common in SAM syndrome and PSO.

Based on the observation that the transcriptional profiles from *Dsg1^–/–^* animals and SAM syndrome shared similarities with PSO (Figure 7, A, B, and D–F) we tested if Dsg1 levels were changed in PSO. PSO samples from lesional skin exhibited a decrease in membrane-associated Dsg1 staining compared with control or nonlesional skin from patients with PSO (Supplemental Figure 7, D and E). Previously, we showed that Dsg1 regulates the stability of the gap junction protein, connexin 43 (Cx43) (36). Consistent with this, membrane levels of Cx43 staining were decreased in areas of reduced Dsg1 in lesional skin from patients with PSO compared with control skin (Supplemental Figure 7, D and E). These observations raise the possibility that downregulation of Dsg1 in PSO has functional consequences for keratinocyte behavior. A reduction of Cx43 levels was also observed in areas with reduced Dsg1 in PF patient epidermis, suggesting that our reported dependence of Cx43 stability on Dsg1 holds true across genetic and autoimmune disease (Supplemental Figure 7, F and G).

### Treatment of SAM syndrome with ustekinumab resulted in clinical improvement.

**Figure 8:**
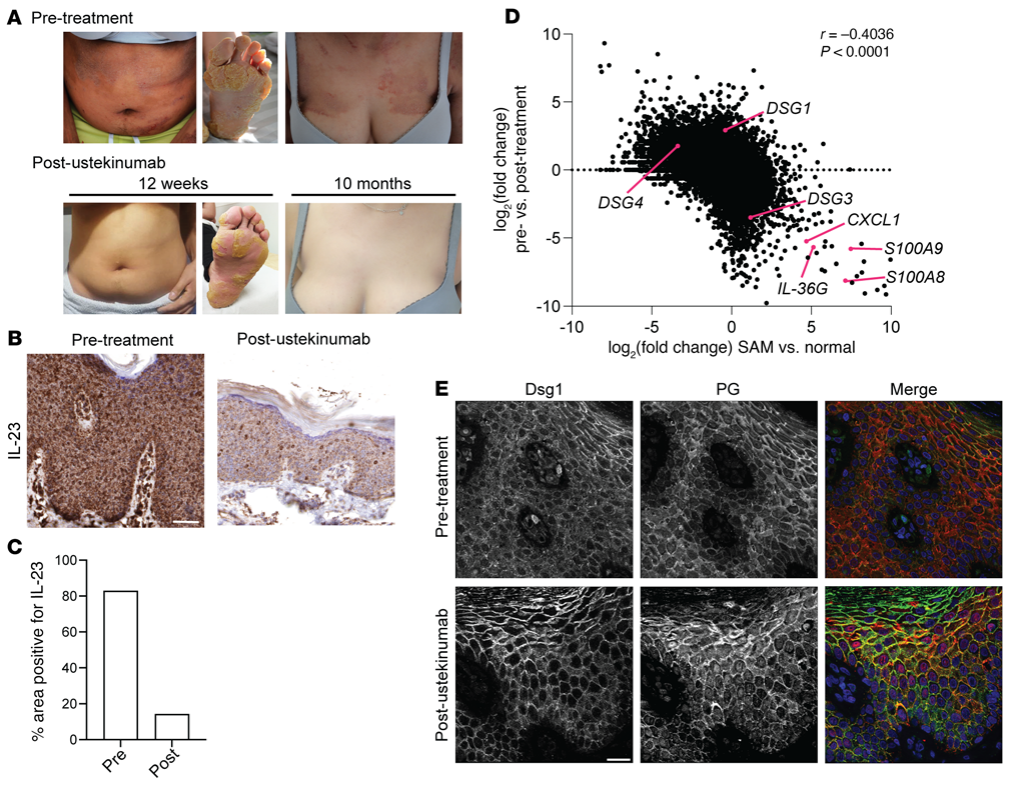
Ustekinumab treatment results in clinical improvement in patients with SAM syndrome. (**A**) Clinical photos of a patient with SAM syndrome (p.Arg887* mutation) before (pre-treatment) and after (post-ustekinumab) 12 weeks and 10 months of treatment with the IL-23/IL-12–blocking antibody ustekinumab. (**B**) IHC staining for IL-23 in the patient with SAM syndrome before and after treatment with ustekinumab for 10 months. Scale bar = 50 μm. (**C**) Analysis of area positive for IL-23 staining in skin biopsies collected before and after ustekinumab treatment (*n =* 1 patient, 4 images analyzed per condition). (**D**) Correlation analysis of control versus SAM lesional RNA-Seq data sets compared with post-ustekinumab RNA-Seq data sets versus lesional pre-ustekinumab treatment shows restoration of both structural and inflammatory gene signatures in the treated sample (*n =* 1 patient, *r* = –0.4036, calculated using Spearman correlation). (**E**) Immunostaining for Dsg1 and plakoglobin (PG) in skin biopsies collected before and after ustekinumab treatment shows restoration of Dsg1 staining following treatment. Scale bar = 20 μm (*n =* 1 patient).

Based upon observations of increased IL-17–driven inflammatory signatures in the skin of patients with SAM syndrome, we tested whether IL-23, which promotes formation of Th17 cells, was elevated in the skin of these patients. We stained skin biopsies from 4 patients with SAM syndrome for IL-23 and observed an increase in IL-23 levels in all cases compared with control (Supplemental Figure 8A). Based on this observation we treated 2 siblings with SAM syndrome, caused by the p.Arg887* Dsg1 mutation, with the IL-12/IL-23–blocking antibody ustekinumab (Supplemental Figure 5). The first patient presented at baseline with erythematous plaques covering 30% of the body surface and severe plantar keratoderma (Figure 8A). Following 12 weeks of treatment, itch was markedly improved, body lesions cleared, and peeling of plantar keratoderma was observed. Improvement continued with 10 months of treatment, including an approximately 4-fold reduction in epidermal IL-23 staining (Figure 8, A–C). The second patient also presented with erythematous plaques covered with fine scales, involving 50% of their body and accompanied by intractable pruritus. Substantial clearing of skin lesions was observed after 12 weeks of treatment with ustekinumab (Supplemental Figure 8B).

To further analyze the effect of ustekinumab in patients with SAM syndrome, we performed RNA-Seq on pre- and posttreatment skin from the first patient (presented in Figure 8A). Comparison of these RNA-Seq data sets revealed a significant negative correlation, suggesting that the overall transcriptional signature in patients with SAM syndrome returns toward normal with ustekinumab treatment (Figure 8D). This signature included a decrease in inflammatory genes such as *CXCL1*, *IL36G*, *S100A9*, and *S100A8*, as well as a decrease in *DSG3* and an increase in *DSG1*. While patients with SAM syndrome have loss of function mutations in Dsg1, we previously showed that cell borders in nonlesional skin retain Dsg1 immunofluorescence, whereas staining in lesional skin is decreased (36). Consistent with the RNA-Seq data showing an increase in *DSG1* mRNA, staining for Dsg1 in the pre- and posttreatment skin from the first patient revealed an increase in Dsg1 levels at cell borders after ustekinumab treatment (Figure 8E). Cx43 was also more organized in the treated sample, and the number of CD3-positive immune cells decreased in the skin upon ustekinumab treatment, in line with the decreased inflammatory signature (Supplemental Figure 8, C and D). These observations demonstrate the clinical relevance of the observed IL-17A response pathways in the transcriptome of patients with SAM syndrome. They also reveal the importance of suppressing inflammation for restoring structural/adhesive proteins important for maintaining epidermal architecture in these genetically susceptible patients.

## Discussion

The epidermis is an immune organ that responds to environmental toxins, pathogens, and mechanical stress through the coordinated activities of both keratinocytes and immune cells (37). Evidence is now emerging that cyto-architectural components in keratinocytes are important contributors to keratinocyte responses to external stimuli. These architectural elements include desmosomes and their associated keratin intermediate filaments. For instance, proliferation-associated keratins K6 and K16/17, which are turned on in response to a variety of stresses and in disorders such as PSO, have special structural roles and also act as alarmins that help stimulate innate and adaptive immunity (38). Intermediate filament anchoring desmosomes appeared during evolution around the time that adaptive immunity developed in jawless fish, and the keratinocyte specific desmosomal cadherin Dsg1 appeared even later when vertebrates became terrestrial and required a barrier suited to a new environment (18). We recently proposed the idea that Dsg1 acts as a sensor of environmental stress by remodeling the secretome upon stress-induced downregulation (16–18). Supporting this, chronic loss of Dsg1, as occurs in SAM syndrome, is associated with chronic inflammatory and allergic disease. The extent to which keratinocyte Dsg1 directly controls these inflammatory and allergic responses and a systematic analysis of genes controlled by Dsg1 has not been explored in an in vivo setting.

Here we show that beyond its essential role in maintaining tissue integrity through desmosomal cell-cell adhesion in granular layers, Dsg1 controls epidermal differentiation and inflammatory gene expression (22). Importantly, the elevation of an inflammatory program occurs during embryogenesis, suggesting that Dsg1 may control these responses independently of any response to environmental stimuli.

Our previous work in an in vitro model of human epidermal morphogenesis demonstrated that in the absence of Dsg1, epidermal differentiation is impaired. This impairment is due to the loss of Dsg1-dependent attenuation of the EGFR/MAPK pathway and consequent failure of expression of genes involved in keratinocyte differentiation (19, 20). Global transcriptomics of skin from the Dsg1-deficient mouse described here showed upregulation of ErbB and MAPK pathways, consistent with results from this human model. Consistent with what was observed in this in vitro human model, protein levels and staining intensity for loricrin are also decreased in *Dsg1^–/–^* animals, and cell shapes are irregular, likely contributing to the observed barrier defects. Comparing the differentiation program over time during development revealed several patterns. In the case of *Dsc1*, gene expression is low at E17.5 in *Dsg1^–/–^* animals but reaches or exceeds the level of *Dsc1* in *Dsg1^+/+^* skin at E18.5, consistent with an observed increase in Dsc1 staining intensity in *Dsg1^–/–^* skin at this time point. The reduced *Dsc1* at E17.5 more closely matches what we previously reported in Dsg1-deficient human organotypic cultures (19). Like in E18.5 embryos, Dsc1 is elevated in SAM syndrome caused by Dsg1 mutations (36). Several other related genes show similar, albeit less pronounced trends as observed for *Dsc1*, whereas some genes, such as *Loricrin*, remain low in the *Dsg1^–/–^* mouse compared with *Dsg1^+/+^* animals at E18.5. It seems likely that there are compensatory responses in some genes, occurring through transcriptional or posttranscriptional mechanisms. Indeed, compensation has been shown to be common in the setting of a genetic mutation, but not in the setting of loss of function through knockdown (39). This is speculated to occur through loss of negative feedback loops, a response that appeared during evolution to make robust biological systems.

We first observed signs of an inflammatory gene signature in *Dsg1^–/–^* mice at E17.5, a time when adhesion and differentiation genes are suppressed compared with *Dsg1^+/+^*mice. The inflammatory signature gained prominence at E18.5 as differentiation was restored. Thus, alterations in epidermal adhesion and architecture occur concomitant with an increased inflammatory signature, making it difficult to determine which happens first during development. Previously, we showed that Dsg1 knockdown in cultured normal human epidermal keratinocytes causes a cell autonomous increase in a cohort of inflammatory cytokines that overlap with those upregulated in the *Dsg1^–/–^* mice (16). Data also suggest that Dsg1 suppresses NFκB/ERBIN-driven cytokine gene expression (14). Together, these data are consistent with a direct, keratinocyte autonomous role for Dsg1 in suppressing inflammatory cytokine expression that could amplify changes due to loss of the physical barrier in vivo.

Patients with SAM syndrome harboring Dsg1 loss of function mutations have severe allergies, and keratinocytes isolated from those patients produce Th2 cytokines (10). Dsg1 deficiency also contributes to an allergic disorder called eosinophilic esophagitis (EOE), which exhibits Th2 skewing (40), and some patients with SAM syndrome exhibit EOE as well (10). In addition, T cells isolated from patients with an endemic form of PF exhibit a Th2-skewed cytokine profile (41). In spite of these indicators of Th2 expression, the upregulated genes in the whole transcriptome analysis of *Dsg1^–/–^* animals and patients with SAM syndrome were enriched for Th17-associated pathways and a response to IL-17A. An increase in the IL-36 pathway was also observed, which has been associated with multiple inflammatory disorders, including PSO where it promotes Th17 skewing through recruitment of Th17 cells, as well as a reduction in the keratinization program (42). While the genetic and antibody-induced disorders share similarities in skin lesion morphology and signs that epidermal differentiation and keratinization programs are altered, the PF skin transcriptome lacks a strong IL-17 inflammatory signature. The potential involvement of NFκB1 and RELA in expression of upregulated inflammatory genes in *Dsg1*^–/–^ mice and SAM, but not PF, is consistent with their well-known role as proinflammatory transcription factors.

The Th17 skewing due to Dsg1 deficiency is reminiscent of recent observations that patients with ichthyosis with various underlying genetic bases, all having previously reported links to AD, showed robust Th17/IL-23 skewing (43, 44). A difference here, however, is that the Th17 inflammatory response is observed before birth in the Dsg1-deficient model, thus supporting a primary role for Dsg1 in the response. In this regard it is interesting to note that pediatric AD populations share certain features of PSO, such as Th17 skewed inflammation, while adults from European and American populations skew more toward Th2 inflammation (23).

Our observations also raise the question of whether Dsg1 loss may be a common factor in PSO and other ichthyoses with Th17 skewing, as is the case in Netherton syndrome, in which Dsg1 is degraded due to loss of LEKTI-1 (lympho-epithelial kazal type related inhibitor type 5) function (45, 46). Dsg1 reduction was observed in a cohort of PSO patient specimens, along with other changes we have previously linked with Dsg1 loss, such as Cx43 mislocalization, consistent with the possibility that Dsg1 loss may contribute to cytokine profiles in this disorder. While we do not yet know how Dsg1 is lost in PSO lesions, we do know that Dsg1 is particularly sensitive to stress-induced downregulation compared with other desmogleins and cadherin family members. Dsg1 expression is reduced in response to certain cytokines such as IL-13 in EOE, where Dsg1 loss has been implicated in contributing to disease pathogenesis (40). Dsg1 loss also occurs in response to bacterial exposure (47) and UV irradiation (17). Dsg1 is a substrate for MMPs and ADAM family proteases, the latter of which are known to contribute to PSO phenotypes through activating EGFR and TNF (48). The observations that Dsg1 loss in utero is sufficient to stimulate a Th17 skewed inflammatory response and Dsg1’s unique sensitivity to loss via extrinsic stressors, suggest that Dsg1 loss may activate a protective response. This is consistent with our recent findings that UV exposure selectively downregulates Dsg1, and conditioned media from Dsg1-deficient keratinocytes stimulates increased melanin secretion in primary melanocytes, a protective response to UV exposure (16, 17).

The distinct IL-17/23 signature present in animals and humans with genetic deficiencies in *Dsg1* prompted us to evaluate the efficacy of the IL-12/IL-23 inhibitor ustekinumab in 2 patients with a defined mutation in *Dsg1*. The observed improvement in the patients’ skin lesions supports the clinical importance of our findings. RNA-Seq data sets from a patient before and after treatment revealed that in addition to suppressing the inflammatory response, ustekinumab restored expression of structural/adhesive proteins important for maintaining epidermal architecture, consistent with previous reports that IL-17A can downregulate differentiation and cornified envelope-related genes (49). These adhesive proteins included the genetic target, Dsg1, which was restored at cell-cell borders in treated patient epidermis. The treated patient’s mutation (p.Arg887*) lacks the terminal region of the Dsg1 C-terminus, removing roughly the same region as an engineered Dsg1 mutant we previously showed supports epithelial sheet integrity in vitro (21). This suggests that eliminating inflammation likely restores adhesion in these genetically susceptible patients.

Our observations are also consistent with previous reports that patients with SAM syndrome caused by a *DSP* mutation responded well to ustekinumab (50) and 2 patients with SAM syndrome caused by *DSG1* mutations responded well to secukinumab (51, 52). These observations also provide a distinction from PF, where the only reports of successful treatment were in a patient with PF and pustular psoriasis, a disease described to benefit from secukinumab (53, 54). Future work to analyze shared and distinct features of Dsg1 deficiency and common skin disorders could provide an opportunity to develop more targeted therapeutic approaches for both rare and common inflammatory disorders. In addition, our work raises the possibility that Dsg1 reduction could be a biomarker for Th17 skewing and taken into consideration when designing therapeutic protocols for skin disorders.

## Methods

### Generation of Dsg1a and Dsg1b exon 2–deleted mouse models.

For details regarding the generation of *Dsg1a* and *Dsg1b* exon 2–deleted mice, see supplemental methods.

### Generation of Dsg1a-c null mice.

Mouse lines harboring the deletion of the approximately 170 kb, desmoglein 1 tandem gene cluster (*Dsg1 c, a, b*) on chromosome 18 of the mouse were generated in the Northwestern University Transgenic and Targeted Mutagenesis Laboratory. Gene editing using CRISPR/Cas9 technology was used to generate the deletion. mRNA Cas9 (GeneArt, Invitrogen A29378) and IVT sgRNAs (*Dsg1c*-L2 TAAATGACCCGGGGATTAGT and *Dsg1b*-R2 GGTTCAGGGAGGCTTCCCGC) were injected at a concentration of 25, 12.5, and 12.5 ng/μL, respectively, into the cytoplasm of single-cell fertilized C57BL/6J zygotes to introduce double strand breaks 5′ (*Dsg1g*-L2-5′) and 3′ (*Dsg1b*-R2) of the genes of the Dsg1 tandem. Microinjected zygotes were transferred into recipient females, and the progeny analyzed for gene editing. One founder with the approximately 170 kb desired deletion was identified, sequence verified, and backcrossed for 10 generations with no change in the phenotypes described here. Both sexes were included in all analyses. Ages of animals used (E15.5–E18.5 and P1) are indicated in the figure legends.

### RNA-Seq expression profiling of Dsg1^–/–^ mice.

RNA for RNA-Seq expression profiling was collected from flash-frozen dorsal skin from E15.5, 16.5, 17.5, and E18.5 mouse embryos using the Quick-RNA miniprep kit (Zymo Research) or RNAeasy kit (Qiagen) following manual homogenization using the Tissue Squisher (Zymo Research) in lysis buffer. cDNA was synthesized using 1 μg RNA using the Superscript III First Strand Synthesis Kit (Life Technologies/Thermo Fisher Scientific). RNA quality was determined using the 2100 Bioanalyzer (Agilent). One microgram RNA from each sample was used for mRNA enrichment using NEBNext Poly(A) mRNA magnetic isolation module (E7490S, New England Biolabs). RNA-Seq libraries were constructed using the purified mRNA samples with NEBNext Ultra II Directional RNA library Prep Kit for Illumina (E7760S, New England Biolabs). The quality of these RNA-Seq libraries was validated using 2100 Bioanalyzer (Agilent), and sequencing was performed by NUseq Core Facility using the Illumina Hiseq4000 (1 × 50 bp) or the Advanced Genomics Core of the Skin Biology Diseases Resource–based Center at the University of Michigan using the Illumina NovaSeq 6000 system (Illumina).

### RNA-Seq expression profiling of SAM syndrome and PF samples.

RNA was isolated from 10 μm sections of formalin-fixed paraffin embedded blocks from 4 lesional samples and 1 nonlesional sample of SAM syndrome skin, 4 healthy control skin samples, 1 before and 1 after treatment skin sample from a patient with SAM syndrome, samples from 7 patients with established PF, and 4 PF-matched control samples. RNA was extracted using the E.N.Z.A. FFPE RNA Kit (Omega Bio-tek). Samples were prepared using the Lexogen 3′ QuantSeq mRNA-Seq Library Prep Kit FWD and sequenced on the Illumina NovaSeq 6000 system.

### Data availability.

RNA-Seq data were submitted to NCBI’s Gene Expression Omnibus repository (GSE189094: E18.5 RNA-Seq data set #2; GSE189095: E15.5–E18.5 RNA-Seq data set #1; GSE189096: patient RNA-Seq data sets; and GSE179162: human single-cell RNA-Seq).

### Additional information on methods and analyses, RNA-Seq data, gene expression, and antibodies.

See supplemental methods for detailed descriptions of immunofluorescence and image acquisition, electron microscopy methods, barrier function assays, RNAscope methods, whole mount staining and analysis, and immunoblot methods. Also see supplemental methods for a detailed description of RNA analysis of mouse tissues and Supplemental Table 9 for sequences of primers used, as well as a detailed description of RNA-Seq data processing and analysis, additional data sets used in this study, and a detailed list of antibodies used in this study. See complete unedited blots in the supplemental material.

### Statistics.

Unless otherwise stated, statistical analysis was performed using 1-way ANOVA, with a Tukey correction for multiple comparisons, with all groups compared in each experiment. *P* less than 0.05 was considered statistically significant in all experiments unless otherwise stated. All data represent mean ± SEM unless otherwise stated.

### Study approval.

All housing, care, and use of animals was handled according to the Northwestern University and CEINGE Institutional Animal Care and Use Committees (protocol ID IS00001419 and Health Ministry protocol 888-2019-PR). *Dsg1^+/+^* and *Dsg^+/–^* mice on a C57BL/6 background were housed in a barrier facility in a temperature-controlled room with a 12-hour light cycle and given ad libitum access to food and water. Mice were mated overnight and separated the following morning to generate timed pregnancies. Embryos were harvested on days E15.5–E18.5 and dorsal skin was collected from the embryo pelts.

Patients with SAM syndrome, originally identified and described in ref. 10, were recruited from the Department of Dermatology, Emek Medical Center (EMC), Afula, Israel, from 2016–2020. Patients or their legal guardians provided informed consent prior to their inclusion in the study, according to a protocol approved by the EMC Institutional Review Board (no. 0086-15) and by the Israel National Committee for Human Genetic Studies in adherence to the Helsinki guidelines. Patients or their legal guardians signed informed consent forms for publication of clinical photos. Clinical follow-up was documented and skin biopsies were obtained from both lesional and nonlesional areas. Pre- and post–ustekinumab treatment biopsies from patients with the p.Arg887* mutation were taken from the lower back. Biopsies for the other 2 mutations were taken from posterior thighs.

Patients with PF, PSO, or AD, and the healthy adults, provided informed consent for skin biopsies. Tissues were anonymized for analysis and collected under IRB no. HUM00087890 at the University of Michigan, and IRB no. STU00009443 at Northwestern University. Biopsies from patients with AD and PSO were taken from extremities (28).

## Author contributions

KJG, LMG, and CM conceived and designed the project. LMG, QRRC, JLK, JAB, GNF, ALH, HEB, MH, SA, JLJ, GU, and ECB performed and analyzed experiments. LMG, KJG, LTD, and RA developed the *Dsg1^–/–^* mouse model. QRRC, LCT, J Kirma, J Kweon, SML, XX, WRS, XB, and JEG performed bioinformatic analysis. PWH, ES, ECB, and JEG provided critical resources. QRRC and KJG drafted the manuscript. LMG, QRRC, KJG, and ALH critically revised the manuscript.

## Supplementary Material

Supplemental data

## Version 1 12/14/2021

In-Press Preview

## Version 2 02/01/2022

Electronic publication
