# Supplementary material for: Translational implications of Th17-skewed inflammation due to genetic deficiency of a cadherin stress sensor
Source: J Clin Invest. 2022 Feb 1;132(3):e144363. doi: 10.1172/JCI144363 (PMC8803337; doi:10.1172/JCI144363)

Supplemental Material

Translational implications of Th17-skewed inflammation due to genetic deficiency of a cadherin stress sensor

Lisa M Godsel, Quinn R Roth-Carter, Jennifer L Koetsier, Lam C Tsoi, Amber L Huffine, Joshua A Broussard, Gillian N Fitz, Sarah M Lloyd, Junghun Kweon, Hope E Burks, Marihan Hegazy, Saki Amagai, Paul W Harms, Xianying Xing, Joseph Kirma, Jodi L Johnson, Gloria Urciuoli, Lynn T Doglio, William R Swindell, Rajeshwar Awatramani, Eli Sprecher, Xiaomin Bao, Eran Cohen-Barak, Caterina Missero, Johann E Gudjonsson, Kathleen J Green

Supplemental Methods

Generation of Dsg1a and Dsg1b Exon 2 deleted mouse model.

To mimic patient mutations which cause severe dermatitis, multiple allergies and metabolic wasting (SAM) Syndrome we deleted exon 2 from *Dsg1a* and *Dsg1b* in the mouse. CRISPR/Cas9 technology was used to generate exon 2 deletions in mouse *Dsg1a* and *Dsg1b* genes using sgRNA recognizing identical intronic sequences flanking exon 2 in *Dsg1a* and *Dsg1b*, but not in *Dsg1c*. Four sgRNA sequences were identified using Chopchop (http://chopchop.cbu.uib.no) and the best combination of sgRNA was selected by preliminary experiments in NIH3T3 cells, sgRNA2-CACCGAGACACATTACTTTGACATG, sgRNA4-CACCGGGAGGAGTAATTATGTCAGG. The selected sgRNA oligonucleotides were subcloned in the BbsI site into pSpCas9(BB)-2A-GFP (PX458, #48138, Addgene) or pSpCas9(BB)-2A-Puro (PX459, #62988 Addgene) vectors. Cloning was verified by Sanger sequencing using the U6F1 primer (TACGATACAAGGCTGTTAGAGAG) that read-through the region of the BbsI site.

Mouse embryonic stem cells (mESC) E14Tg2A.4 were cultured in GMEM medium supplemented with 12% FBS (Hyclone CHA30070L), 2 mM Glutamine, 1 mM Sodium Pyruvate, 1x non-essential amino acids, 50 µM β-mercaptoethanol, 1000 U/mL ESGRO Leukemia inhibitory factor (Sigma). mESC cells were transfected with both vectors using Lipofectamine 2000 (Gibco). 10,000 cells were plated in 10 cm^2 plates. After 30 hours cells were selected with 3 µg/ml puromycin for 48 hourrs. Cells surviving selection were analyzed for GFP expression. After 10 days, 171 clones were verified for the absence of Cas9 integration and homozygosity for exon 2 in both *Dsg1a* and *b.* One clone was used for blastocyst injections at the Institute of Genetics and Biophysics (IGB, Naples, Italy). Ten chimeras were born with skin erosions and did not survive.

Exon 2 deletion was determined by PCR on genomic DNA using the following specific oligonucleotides: *Dsg1a* forward (TGACCCTCAGTGCAATACAAAA), *Dsg1a* reverse (CTGGGCATGTTGAATCCTGTAA), *Dsg1b* forward (TGAACACCCATTACATGCTTCC), *Dsg1b* reverse (TTCGAATGAAGAGGTGCCTTTA).

Immunofluorescence, immunohistochemistry, and image acquisition.

Dorsal mouse skin, SAM patient and healthy control skin samples were either fixed in 10% formalin, embedded in paraffin blocks and cut into 4 µm thick sections, or embedded in Optimal Cutting Temperature compound (OCT, Tissue-Tek) and cut into 4-5 µm sections. For immunostaining, paraffin sections were baked at 60°C overnight and de-paraffinized using xylene. Samples were then rehydrated through a series of ethanol and PBS dips, and slides were permeabilized in 0.5% Triton X-100 in PBS. Antigen retrieval was performed by incubation in 0.01 M citrate buffer at 95°C for 15 minutes. Sections were blocked in blocking buffer (1% BSA, 2% normal goat serum in PBS) for 60 minutes at 37°C. Samples were then incubated in primary antibody at 4°C overnight, followed by incubation in secondary antibody

for 1 hour at 37°C. OCT samples were allowed to warm to room temperature and were fixed either with 4% paraformaldehyde or 100% anhydrous methanol. Staining for OCT samples followed the same method as paraffin samples, excluding the antigen retrieval steps. Images were acquired using an AxioVison Z1 system (Carl Zeiss) with Apotome slide module, an AxioCam MRm digital camera, and either a 20x (0.8 NA Plan-Apochromat) or 40x (1.4 NA, Plan-Apochromat, oil objective). Image analysis was carried out using ImageJ software. For immunohistochemistry of SAM Syndrome patient samples, the I-View DAB detection kit (Ventana, Roche, San Jose) was used according to the manufacturer's instructions.

For exon 2 deletion animals immunohistochemistry was performed with the R.T.U. VECTASTAIN Universal Elite ABC Kit (Vector Laboratories PK-7200), following the manufacturer's instructions. Detection was performed with DAB Peroxidase Substrate (Vector Laboratories SK-4100). Tissue was counterstained with hematoxylin (Hematoxylin QS; Vector H-3404). Images were acquired using an Axioskop 2 Plus (Carl Zeiss) and AxioCam color digital camera 20x (0.5 NA, Plan-Neofluar) objective.

RNA analysis of mouse tissues.

Mouse C57BL/6 organs were dissected and total RNA was extracted using TRIzol reagent (Invitrogen). cDNA was synthesized using SuperScript Vilo (Invitrogen). Reverse transcription qRT-PCR was performed using the SYBR Green PCR master mix in an ABI PRISM 7500 (Applied Biosystems). Levels of the target genes were quantified using specific oligonucleotide primers and normalized to actin.

RNA for qRT-PCR experiments was collected from flash frozen dorsal skin of E18.5 mice and isolated using the Quick-RNA miniprep (Zymo Research) following manual homogenization using the Tissue Squisher (Zymo Research) in lysis buffer. cDNA was synthesized using 1 μg of RNA using the Superscript III First Strand Synthesis Kit (Life Technologies/Thermo Fisher). Quantitative PCR was performed on the QuantStudio 3 instrument (Thermo Fisher), using SYBR Green PCR master mix (Thermo Fisher). Relative mRNA levels were calculated using the ΔΔCT method normalized to GAPDH. Primer sequences are listed in Supplemental Table 9.

Antibodies.

Antibodies used in this study include: mouse anti-Dsg1 (P124, 651111, Progen; 27B2, 32-6000, Thermo Fisher; 4B2(1), B-11, sc-137164, Santa Cruz Biotechnology), mouse anti-Dsg3 (D219-3, MBL International Corp.), anti-Dsc1 (sc-39859, Santa Cruz Biotechnology), mouse anti-Ecad (610181, BD Biosciences, anti-Cx43 (AB1728, EMD Millipore), chicken anti-PG 1407 (Aves Laboratories), rabbit anti-transglutaminase (sc-25786, Santa Cruz Biotechnology), IL-23p19 (511201, Biolegend), rat anti-S100A9 (ab105472, Abcam), rabbit anti-S100A9 (242945, Abcam), rabbit anti-CD3e (MA1-90582, ThermoFisher Scientific) mouse anti-desmoplakin (11-5F, 91121236-1VL, Sigma Aldrich), mouse anti-actin (C4, MAB1501, Millipore Sigma), rabbit anti-GAPDH (G9545, Millipore Sigma), AlexaFluor 568/647-conjugated goat anti-mouse and -rabbit secondary antibodies (ThermoFisher Scientific) were used in

immunofluorescence studies. Immunoblot analyses include use of peroxidase-conjugated anti-mouse and -rabbit secondary antibodies (SeraCare Life Sciences). The following antibodies were a gift from J Segre (National Human Genome Research Institute, National Institutes of Health): rabbit anti-loricrin, rabbit anti-involucrin.

RNAscope™.

RNA *in situ* hybridization was completed using RNAscope technology with the ACDBio RNAscope 2.5HD Reagent Kit - Brown (ACDBio, Hayward, CA). FFPE tissues were sectioned at 4 μm and hybridized with probes against *Dsg1a* (ACDBio, 84861) or *Dsg3* (ACDBio, 464301). The protocol was followed according to manufacturer's instructions and hybridized RNA was detected with DAB and counterstained with Gill's hematoxylin.

Immunoblot analysis of proteins.

Immunoblots for exon 2 knockout mice were performed as follows. Skin samples were lysed with lysis buffer (6% SDS, 0.125M Tris-HCl pH 6.8, 1x Protease Inhibitor, 1x Phosphatase Inhibitor and 1x PMSF) supplemented with 20% β-mercaptoethanol and separated by SDS-PAGE. Transferred blots were then incubated with the Dsg1 B-11 antibody and the anti-actin antibody for 2 hours at room temperature, then incubated with HRP conjugated secondary for 1 hour at room temperature.

All other immunoblots were performed as follows. Lysates were collected from E18.5 dorsal skin by manual homogenization using the Tissue Squisher (Zymo Research) in urea sample buffer (8 M urea, 1% SDS, 60 mM Tris (pH 6.8), 5% β-mercaptoethanol, 10% glycerol), sonicated and centrifuged. Samples were separated by SDS-PAGE and transferred to nitrocellulose membranes. Membranes were blocked with 5% milk in PBS, incubated with primary antibody overnight at 4°C, and secondary antibody conjugated to HRP for 1 hour at room temperature. Proteins were imaged using chemiluminescence on the Odyssey FC imaging system (Licor) or exposed to film. Densitometry values were analyzed using ImageStudio software (Licor) and normalized to GAPDH or actin.

Whole mount imaging and analysis.

Dorsal skin was harvested from E18.5 mice, and either fixed immediately for 2 hours in 4% paraformaldehyde or incubated with 2.4 U/mL dispase in PBS at 37°C for 1 hour. The epidermis was peeled from the dermis and fixed for 15 minutes with 4% paraformaldehyde. Samples were blocked in blocking solution (5% normal goat serum, 1% Triton X-100, in PBS) overnight at 37°C. Samples were then incubated with Phalloidin-647 (ThermoFisher Scientific) diluted in blocking solution overnight at 37°C and then mounted onto glass slides with Prolong Gold (Life Technologies). Z-stack images (z-step size of 1.5 μm) were taken on a Nikon A1R confocal laser scanning microscope with two PMT detectors and two GaAsP detectors using a 40x objective (1.0 NA, Plan Apochromat, Oil), controlled by NIS Elements software (Nikon). Analysis of cell circularity was performed using ImageJ software on phalloidin stained images.

Additional gene expression datasets.

The fold change signature of *Dsg1*$^{-/-}$ vs. *Dsg1*$^{+/+}$ skin was compared to 36 others generated from microarray experiments comparing psoriasis (PSO) or atopic dermatitis (AD) lesions to normal or uninvolved human skin (Figure 7). Genome-wide fold-change estimates were calculated from each PSO/AD vs. normal/uninvolved comparison as described previously (2). Mouse genes were paired with their human orthologues based on the Homologene database (https://www.ncbi.nlm.nih.gov/homologene), creating human-mouse orthologous gene pairs. Spearman's *rho* statistic was calculated for each comparison with p-values calculated based on the asymptotic t approximation. To identify genes robustly elevated by PSO and AD, we calculated meta-signatures by averaging fold-change estimates across the subset of comparisons that used the same Affymetrix Human Genome Plus 2.0 microarray platform (n = 11, PSO; n = 10, AD). Human genes without a mouse orthologue were excluded from this analysis. Based on the composite meta-signature, we identified the 100 genes most strongly increased by PSO and AD (i.e., highest average fold-change) and the 100 genes most strongly decreased by PSO and AD (i.e., lowest average fold-change). We then evaluated cumulative overlap between these 100 genes and the list of corresponding mouse genes ranked according to *Dsg1*$^{-/-}$/*Dsg1*$^{+/+}$ fold-change.

RNA-seq data processing and analysis.

Quality control and adaptor trimming were performed on sequence reads from the RNA-seq data. STAR alignment (3) was used to align reads to the reference (GRCh37 for human and mm10 for mouse samples)(4). HTSeq was used for gene quantification and DESeq2 (5) was used for normalization and differential expression analysis.

For the comparison between the different datasets (e.g. *Dsg1*$^{-/-}$ vs SAM), only genes that are common were considered in the analysis. Mouse genes were paired with their human orthologue based on the Homologene database (https://www.ncbi.nlm.nih.gov/homologene), creating human-mouse orthologous gene pairs. To identify the cytokine signature of each skin condition, we took the genes induced by cytokines in human keratinocytes, and computed the enrichment among the top 500 most significant genes upregulated in the corresponding skin condition using the hypergeometric test. Data are plotted as Observed/Expected ratio for enrichment in cytokine response as a function of the adjusted p value, with adj p value < 0.05 considered statistically significant. The data set for the keratinocytes treated with cytokines was previously published in Tsoi et al. 2019 (6). Functional enrichment analysis was performed using Metascape (metascape.org) using the Gene Ontology (GO) pathways, and Kyoto Encyclopedia of Genes and Genomes (KEGG) pathways. Pathways were considered statistically significant with $p < 0.05$. To identify changes in gene expression of genes involved with specific stages of keratinocyte differentiation a single-cell RNA-seq data set was used to identify genes expressed in basal, differentiated, or keratinized

keratinocytes in an unbiased manner. Expression levels of these genes from the different datasets (i.e., SAM Syndrome and PF) were graphed.

Transmission electron microscopy.

Dorsal skin from E18.5 embryos were processed for conventional electron microscopic analysis as described in (7). Briefly, dorsal skin was cut into pieces and fixed in 0.1 M cacodylate buffer pH 7.3 containing 2% PFA and 2.5% glutaraldehyde overnight. Tissues were postfixed in 2% osmium tetraoxide followed by 2% uranyl acetate. Tissues were dehydrated in ascending grades of ethanol, infiltrated with propylene oxide and embedded in Embed 812 resin, cured overnight at 60°C. Tissues were ultrathin sectioned with a Leica Ultracut UC6 ultramicrotome with a diamond knife and collected on formvar coated copper mesh grids. Sections on grids were stained in 3% uranyl acetate followed by Reynolds lead citrate solution. Grids were rinsed briefly in 0.02 M NaOH, followed by distilled water and air dried. Stained sections were viewed and photographed using an FEI Tecnai Spirit G2 transmission electron microscope.

Skin barrier toluidine blue assay.

For outside-in barrier testing, E18.5 embryos were sacrificed and rinsed in PBS followed by dehydration immersion steps of 25%, 50%, 75%, 100%, 75%, 50%, 25% methanol. After the dehydration steps the embryos were rehydrated in PBS and immersed in 1-5% toluidine blue for up to 10 minutes and washed several times with PBS.

Measuring transepidermal water loss (TEWL).

A Tewameter TM300 system (Courage + Khazaka electronic GmbH) fitted with a small animal adapter was used to measure water evaporation from the skin surface and to quantify epidermal permeability/barrier function of P1 pups over 5 hours on the dorsal back. Measurements were recorded when TEWL readings were stabilized at approximately 45 seconds after the probe was placed on the skin and readings were averaged from 5 readings per time point.

Supplemental Table 1

Upregulated Genes in the *Dsg1*$^{-/-}$ mouse, PSO and SAM Syndrome										
Gene	*Dsg1*$^{-/-}$ #1 FC	*Dsg1*$^{-/-}$ #1 FDR	*Dsg1*$^{-/-}$ #2 FC	*Dsg1*$^{-/-}$ #2 FDR	PSO FC	PSO FDR	AD FC	AD FCR	SAM FC	SAM FDR
COX7B	0.14	0.84	1.06	0.0711	1.09	3.00E-18	0.45	0.00206	1.84	1.91E-05
DBI	0.45	0.241	1.03	0.0568	1.2	5.00E-18	0.56	7.35E-05	1.14	1.70E-05
GBP6	3.57	0.089	1.48	0.232	3.45	1.56E-20	0.45	0.127	2.41	0.0647
GJB2	1.25	2.82E-08	0.3	0.55	4.98	1.47E-32	2.97	1.44E-16	3.65	0.00365
IFI27	1.2	0.274	1.17	0.0136	5.68	8.79E-25	4.51	2.23E-20	1.94	1.40E-10
KRT16	3.54	2.40E-10	3.5	0.00346	6.29	9.22E-27	5.77	3.26E-21	5.28	0.00315
KRT6A	3.1	0.0292	1.41	0.0673	5.5	8.64E-24	4.67	1.51E-17	3.54	0.0668
PLA2G4E	1.27	1.63E-05	0.4	0.381	2.06	1.75E-26	1.17	2.14E-18	1.28	0.0601
PRSS27	1.51	0.0949	0.15	0.885	4.37	3.86E-30	2.87	3.89E-17	4.2	0.00387
S100A8	1.8	0.0119	3.23	3.47E-18	9.11	6.43E-30	6.55	1.52E-22	7.17	1.25E-05
S100A9	1.8	0.0135	2.97	3.46E-11	9.16	1.76E-29	6.73	1.05E-23	7.5	8.48E-08
SAT1	-0.19	0.494	1.06	0.0577	1.34	5.27E-15	0.5	1.06E-05	1.74	0.0033
SLPI	3.34	9.25E-06	2.03	0.0117	1.18	3.11E-10	-0.02	0.909	2.51	9.30E-05
SPRR1B	3.09	7.06E-09	2.16	0.12	4.33	7.06E-31	3.55	2.06E-19	4.1	0.00045
TMEM45B	2.75	0.184	1.81	0.0593	2.32	5.17E-31	1.73	1.01E-19	2.4	0.000385
TMPRSS11D	2.17	0.0947	2.86	0.484	7.16	1.97E-19	2.41	1.87E-08	4.47	0.0386

Supplemental Table 2

Upregulated genes in the *Dsg1*$^{-/-}$ mouse and PSO, but not in SAM Syndrome										
Gene	*Dsg1*$^{-/-}$ #1 FC	*Dsg1*$^{-/-}$ #1 FDR	*Dsg1*$^{-/-}$ #2 FC	*Dsg1*$^{-/-}$ #2 FDR	PSO FC	PSO FDR	AD FC	AD FCR	SAM FC	SAM FDR
APOL6	1.95	0.343	1.73	0.0577	2.62	1.39E-27	1.62	5.34E-18	-0.22	0.818
ARC	2.47	0.0223	0	0.998	1.48	1.51E-09	1.51	6.24E-06	1.62	0.676
CAMP	-0.08	0.952	1.46	0.0977	3.07	2.56E-13	2.1	5.53E-06	-1.57	0.89
CASP4	-0.02	0.988	1.25	0.0315	1.06	4.71E-21	0.53	8.72E-07	0.84	0.133
CCL8	0.3	0.92	2.24	0.0597	2.06	6.89E-07	1.82	2.50E-06	-2.14	0.398
CCR1	0.23	0.694	1.91	2.01E-12	1.08	1.53E-05	1.6	1.323-08	0.11	0.96
CCR2	-0.07	0.918	1.83	9.79E-12	1.22	1.24E-09	1.59	2.83E-10	0.74	0.819
CCR5	0.67	0.151	1.89	5.86E-14	2.11	5.29E-17	1.95	3.53E-14	2.36	0.403
CIB2	0.78	0.368	1.13	0.0342	1.62	5.18E-19	1.21	3.59E-11	0.89	0.653
CLEC4C	0.15	0.838	1.23	0.0335	2.6	2.50E-12	1.91	5.00E-07	-0.84	0.932
CLEC4D	0.99	0.0581	2.49	1.51E-08	2.33	5.22E-10	0.45	0.23	-4.51	0.0123
CLEC4E	0.69	0.542	2.9	3.97E-12	1.72	1.00E-04	0.38	0.318	1.28	0.793
CLEC7A	0.43	0.569	2.57	1.73E-12	3.71	4.78E-34	2.74	1.84E-24	0.4	0.792
CXCL2	3.1	0.115	2.47	0.0086	2.92	2.64E-13	1.8	8.42E-07	0.9	0.844
CXCL3	4.14	0.0351	0.86	0.683	1.66	2.94E-06	1.02	0.00864	1.01	0.901
CXCL6	6.55	0.0281	3.4	0.066	3.56	2.79E-12	2.84	2.11E-06	2.17	0.813
CXCR2	2.39	0.00123	1.75	0.00413	2.33	2.62E-24	0.28	0.158	0.76	0.636
DHRS9	2.63	0.099	-0.36	0.67	2.15	3.50E-12	1.23	1.01E-06	1.24	0.428
DNASE1L3	1.16	0.0177	0	0.998	2.86	9.92E-27	2.19	4.37E-17	1.69	0.214
DSG3	2.32	1.78E-36	0.48	0.505	2.16	7.40E-26	1.99	1.01E-19	1.2	0.106
EPGN	1.01	0.0896	1.64	0.00488	4.58	1.14E-20	3.01	1.91E-06	1.62	0.524
EPSTI1	0.13	0.886	1.07	0.0568	3	6.14E-19	2.11	2.97E-12	-2.1	0.0359
FOSL1	1.27	0.00472	-0.36	0.577	2.59	2.28E-20	3.33	1.33E-10	3.1	0.229
FPR1	1.11	0.205	1.98	1.28E-06	2.16	2.77E-11	1.27	2.27E-05	3.03	0.253
FPR2	1.08	0.182	1.77	0.000412	1.4	4.96E-05	0.13	0.815	0.06	0.998
G0S2	1.01	0.423	1.17	0.0368	1.42	0.00468	1.02	0.0318	-0.44	0.901
GK	0.33	0.906	1.91	0.0745	1.79	9.53E-19	0.5	0.00051	0.48	0.785
GNGT2	-0.22	0.565	1.12	0.0759	1.02	0.000348	0.85	0.00363	-1.94	0.843
GZMA	0.45	0.736	1.14	0.0923	3.18	1.67E-19	1.7	3.55E-07	0.85	0.55
IL1B	1.6	0.0949	3.2	1.44E-10	2.92	1.01E-13	0.7	0.0426	0.7	0.873
ISG15	-0.09	0.905	1.25	0.0611	3.58	1.97E-13	1.88	5.01E-07	0.51	0.895
KLK6	2.24	0.0114	1.99	0.032	4.78	2.69E-17	2.74	3.35E-08	0.49	0.91
LAIR1	0.38	0.658	1.2	0.0153	1.06	1.10E-07	0.79	0.000198	0.35	0.864
LCN2	2.14	0.0156	1.07	0.0781	5.88	1.06E-19	2.68	2.14E-12	1.73	0.235
MEFV	0.17	0.946	3.38	0.00137	2.3	1.86E-13	0.72	0.0166	-5.03	0.0923
MMP9	1.32	0.0568	1.24	0.0144	2.97	5.85E-21	2.01	4.85E-11	-0.99	0.614
MX1	-0.24	0.775	1.04	0.0965	3.87	1.40E-22	2.25	9.88E-12	1.36	0.146
NABP1	0.54	0.363	1.19	0.0332	1.71	4.68E-23	1.22	1.37E-13	0.76	0.45
NEURL3	0.28	0.47	1.06	9.74E-05	1.29	0.000103	1.55	8.76E-07	-0.13	0.998
OLR1	2.65	0.0872	-0.5	0.864	3.18	3.95E-14	1.32	0.00164	-0.69	0.949
PLAC8	0.07	0.935	1.53	0.00146	1.27	6.95E-05	0.8	0.00957	-1.8	0.351
PRR9	0.57	0.735	2.03	0.000224	2.33	2.58E-05	2.23	0.000772	4.71	0.154
PSMB9	-0.06	0.926	1.1	0.038	1.06	6.11E-13	1.05	2.47E-10	0.29	0.821
S100A1	1.1	0.381	1.18	0.00806	1.37	5.66E-08	0.9	0.0014	0.74	0.949
SELE	0.44	0.588	1.58	0.00337	1.99	1.97E-12	2.41	1.03E-15	2.49	0.35
SELL	0.68	0.275	1.97	4.78E-06	1.23	3.15E-09	1.24	2.03E-06	-0.23	0.945
SLC5A8	1.86	1.72E-05	0.61	0.384	1.98	3.95E-05	0.97	0.026	-1.4	0.901
SPRR1A	1.72	0.024	1.75	0.041	4.22	3.71E-24	3.85	8.74E-15	2.19	0.132
STEAP4	1.12	0.0167	0.74	0.123	2.5	1.70E-18	1.88	1.67E-10	0.65	0.655
TIMM8B	-0.3	0.433	1.04	0.0868	1.06	2.81E-18	0.5	0.000848	0.84	0.107
TNFSF10	0.29	0.68	1.19	0.0016	1.87	1.83E-26	1.29	2.85E-13	0.55	0.259
TREM1	1.47	0.0836	1.82	0.0136	2.54	7.01E-10	1.21	0.00599	-0.69	0.949
TRIM10	1.16	0.0532	0.34	0.655	4.04	1.71E-23	3.63	3.60E-18	2.79	0.232
ZBP1	0.35	0.732	1.31	0.0344	3.28	1.93E-12	1.31	3.95E-05	-1.16	0.873

Supplemental Table 3

Downregulated Genes in the *Dsg1*$^{-/-}$ mouse, PSO and SAM Syndrome										
Gene	*Dsg1*$^{-/-}$ #1 FC	*Dsg1*$^{-/-}$ #1 FDR	*Dsg1*$^{-/-}$ #2 FC	*Dsg1*$^{-/-}$ #2 FDR	PSO FC	PSO FDR	AD FC	AD FCR	SAM FC	SAM FDR
AEBP1	-0.32	0.387	-1.09	0.0759	-1.22	4.92E-09	-0.23	0.237	-1.04	0.0477
AFF2	0.26	0.744	-1.13	0.0657	-1.29	2.19E-07	-1.24	7.08E-08	-3.64	0.0141
CDH7	-1.08	0.0452	0.9	0.134	-1.66	6.58E-06	-0.87	0.0342	-5.09	0.0968
CNTN6	-1.05	0.0894	-0.3	0.64	-1.17	0.00558	-1.02	0.0151	-4.64	0.0185
COL24A1	0.12	0.838	-1.06	0.043	-1.29	1.48E-10	-0.6	0.00105	-3.33	0.00125
DCX	-0.37	0.574	-1.02	0.0657	-1.23	0.0013	-0.39	0.404	-4.06	0.000739
ERBB4	-0.13	0.875	-1.81	0.0586	-5.26	2.21E-21	-3.17	8.38E-15	-3.62	0.00405
FAT4	-0.11	0.868	-1.08	0.0891	-1.33	1.73E-10	-0.63	0.000453	-1.8	0.0292
FRAS1	0.1	0.803	-1.08	0.0692	-1.28	6.49E-08	-1.68	1.63E-12	-3.18	0.0102
GLI2	0.24	0.466	-1.05	0.0582	-1.92	8.31E-12	-0.44	0.0358	-1.85	0.0201
HIF3A	-0.24	0.641	-1.15	0.0883	-2.42	1.46E-09	-0.96	0.00122	-2.27	0.0265
PAPPA2	-0.61	0.487	-1.26	0.0442	-2.19	4.02E-07	-1.21	0.000406	-4.83	0.000439
SRGAP1	0.21	0.567	-1.17	0.0759	-1.44	1.36E-13	-0.09	0.565	-1.26	0.292
SSC5D	0.15	0.843	-1.01	0.0842	-2.81	1.48E-16	-1.15	4.79E-06	-1.02	0.0805
TENM2	-0.21	0.838	-1.46	0.0723	-2.04	3.59E-20	-1.19	3.39E-09	-1.48	0.00969
ZNF385B	-0.59	0.0583	-1.03	0.0249	-3.47	1.08E-12	-2.14	2.99E-08	-3.64	0.0122
ZNF652	0.11	0.759	-1.02	0.0781	-1.25	1.48E-17	-0.64	1.32E-09	-1.26	0.02

Supplemental Table 4

Downregulated Genes in the *Dsg1*$^{-/-}$ mouse and PSO, but not in SAM Syndrome										
Gene	*Dsg1*$^{-/-}$ #1 FC	*Dsg1*$^{-/-}$ #1 FDR	*Dsg1*$^{-/-}$ #2 FC	*Dsg1*$^{-/-}$ #2 FDR	PSO FC	PSO FDR	AD FC	AD FCR	SAM FC	SAM FDR
ASTN2	-0.3	0.5	-1.13	0.0169	-1.31	1.18E-06	-0.6	0.0138	-1.17	0.817
C5orf46	-1.78	7.93E-07	-1.3	0.567	-3.4	1.66E-21	-3.68	8.30E-24	1.14	0.42
CACNA1G	-0.19	0.744	-1.18	0.0472	-1.82	4.92E-16	-0.88	3.57E-06	-0.35	0.949
CACNA1H	-0.36	0.426	-1.04	0.0723	-5.14	5.19E-10	-3.51	4.87E-08	-2.65	0.792
CAPN11	-2.26	0.089	-1.87	0.119	-1.89	1.07E-09	-0.98	0.000197	-1.34	0.609
CLDN2	-0.63	0.0867	-1.01	0.00308	-1.08	3.69E-05	-0.34	0.134	-2.21	0.758
CNTNAP3	-1.69	0.0884	-1.01	0.189	-1.88	3.65E-14	-0.73	0.0012	-1.49	0.66
COL1A1	-0.3	0.518	-1.44	0.0838	-1.11	5.62E-05	-0.29	0.314	-0.4	0.788
COL5A1	-0.12	0.812	-1.32	0.0654	-1.02	1.43E-08	-0.22	0.184	-0.26	0.799
CREB3L1	-0.07	0.886	-1.12	0.0708	-1.83	3.54E-08	-0.42	0.111	-0.67	0.504
DCHS1	-0.14	0.767	-1.1	0.0865	-1.34	1.26E-11	-0.54	0.000632	-0.7	0.605
ELFN2	0.48	0.686	-1.61	0.0997	-2.69	1.11E-14	-1.71	6.35E-09	0.6	0.957
ESPN	-0.28	0.471	-1.13	0.0867	-1.21	5.48E-12	0.02	0.896	-0.99	0.528
FRMPD4	-1.22	0.0842	-0.85	0.215	-1.28	0.000283	-0.55	0.125	-1.57	0.89
GPC6	-0.09	0.893	-1.49	0.00563	-2.36	2.34E-12	-0.91	0.000813	-1.57	0.171
HKDC1	-0.81	0.377	-1.19	0.078	-1.03	0.00862	0.12	0.741	-1.69	0.819
IGFBP5	-0.09	0.883	-1.37	0.0462	-2.4	1.39E-20	-1.32	6.80E-13	-0.81	0.206
LCE5A	-2.51	0.0718	-2.79	0.099	-2.97	6.84E-18	-4.02	1.84E-24	-0.91	0.612
MAP1A	-0.09	0.872	-1.21	0.0617	-2.01	8.41E-16	-0.78	3.14E-05	-0.98	0.247
MAP1B	-0.11	0.801	-1.36	0.0329	-1.08	5.95E-10	-0.62	1.54E-05	-0.9	0.0293
NFIC	0.11	0.8	-1.14	0.0677	-1.21	1.07E-14	-0.33	0.00996	-0.36	0.427
NFIX	0.14	0.687	-1.18	0.0865	-1.09	7.96E-20	-0.67	6.89E-11	-0.5	0.166
NPTXR	-0.16	0.786	-1.35	0.053	-1.8	1.74E-13	-0.74	1.36E-05	1.34	0.641
NYNRIN	0.21	0.656	-1.16	0.0911	-1.2	1.19E-16	-0.76	4.37E-10	0.16	0.949
PADI2	-0.17	0.837	-1.52	0.0883	-1.36	2.32E-06	0.62	0.0297	-1.32	0.371
PHYHIPL	-0.28	0.792	-1.64	0.0781	-1.98	2.53E-12	-0.97	4.03E-05	-2.04	0.444
PIRT	-1.11	0.262	-1.19	0.0989	-1.29	0.000411	-0.23	0.59	-2.29	0.819
PLXNA4	0	0.997	-1.17	0.0657	-2.52	9.09E-25	-1.41	8.25E-16	-1.35	0.136
SCUBE1	0.09	0.848	-1.25	0.0574	-3.32	4.31E-15	-2.04	2.22E-10	-1.25	0.896
TNS1	0.3	0.5	-1.05	0.0839	-1.08	6.32E-15	-0.44	0.00102	-0.63	0.321
TRIM9	-0.81	0.105	-1.41	0.0439	-1.27	7.30E-11	-0.48	0.0125	-0.84	0.898
WNK2	0.25	0.477	-1.34	0.0787	-3.03	1.25E-17	-1.06	5.99E-07	-1.85	0.124

Supplemental Table 5

Upregulated Genes in the *Dsg1*$^{-/-}$ mouse, AD and SAM Syndrome										
Gene	*Dsg1*$^{-/-}$ #1 FC	*Dsg1*$^{-/-}$ #1 FDR	*Dsg1*$^{-/-}$ #2 FC	*Dsg1*$^{-/-}$ #2 FDR	PSO FC	PSO FDR	AD FC	AD FCR	SAM FC	SAM FDR
GJB2	1.25	2.82E-08	0.3	0.55	4.98	1.47E-32	2.97	1.44E-16	3.65	0.00365
IFI27	1.2	0.274	1.17	0.0136	5.68	8.79E-25	4.51	2.23E-20	1.94	1.40E-10
KRT16	3.54	2.40E-10	3.5	0.00346	6.29	9.22E-27	5.77	3.26E-21	5.28	0.00315
KRT6A	3.1	0.0292	1.41	0.0673	5.5	8.64E-24	4.67	1.51E-17	3.54	0.0668
PLA2G4E	1.27	1.63E-05	0.4	0.381	2.06	1.75E-26	1.17	2.14E-18	1.28	0.0601
PRSS27	1.51	0.0949	0.15	0.885	4.37	3.86E-30	2.87	3.89E-17	4.2	0.00387
S100A8	1.8	0.0119	3.23	3.47E-18	9.11	6.43E-30	6.55	1.52E-22	7.17	1.25E-05
S100A9	1.8	0.0135	2.97	3.46E-11	9.16	1.76E-29	6.73	1.05E-23	7.5	8.48E-08
SPRR1B	3.09	7.06E-09	2.16	0.12	4.33	7.06E-31	3.55	2.06E-19	4.1	0.00045
TMEM45B	2.75	0.184	1.81	0.0593	2.32	5.17E-31	1.73	1.01E-19	2.4	0.000385
TMPRSS11D	2.17	0.0947	2.86	0.484	7.16	1.97E-19	2.41	1.87E-08	4.47	0.0386

Supplemental Table 6

Upregulated genes in the *Dsg1*$^{-/-}$ mouse and AD, but not in SAM Syndrome										
Gene	*Dsg1*$^{-/-}$ #1 FC	*Dsg1*$^{-/-}$ #1 FDR	*Dsg1*$^{-/-}$ #2 FC	*Dsg1*$^{-/-}$ #2 FDR	PSO FC	PSO FDR	AD FC	AD FCR	SAM FC	SAM FDR
APOL6	1.95	0.343	1.73	5.77E-02	2.62	1.39E-27	1.62	5.34E-18	-0.22	0.818
ARC	2.47	0.0223	0	9.98E-01	1.48	1.51E-09	1.51	6.24E-06	1.62	0.676
CA4	1.72	0.131	1.26	0.00196	0.55	0.184	1.41	1.13E-05	0.61	0.955
CAMP	-0.08	0.952	1.46	9.77E-02	3.07	2.56E-13	2.1	5.53E-06	-1.57	0.89
CCL8	0.3	0.92	2.24	5.97E-02	2.06	6.89E-07	1.82	2.5E-06	-2.14	0.398
CCR1	0.23	0.694	1.91	2.01E-12	1.08	1.53E-05	1.6	1.32E-08	0.11	0.96
CCR2	-0.07	0.918	1.83	9.79E-12	1.22	1.24E-09	1.59	2.83E-10	0.74	0.819
CCR5	0.67	0.151	1.89	5.86E-14	2.11	5.29E-17	1.95	3.53E-14	2.36	0.403
CD300LB	0.19	0.863	1.61	0.0042	0.99	0.00442	1.17	0.000603	0.29	0.979
CIB2	0.78	0.368	1.13	3.42E-02	1.62	5.18E-19	1.21	3.59E-11	0.89	0.653
CLEC4C	0.15	0.838	1.23	3.35E-02	2.6	2.50E-12	1.91	5.00E-07	-0.84	0.932
CLEC7A	0.43	0.569	2.57	1.73E-12	3.71	4.78E-34	2.74	1.84E-24	0.4	0.792
CXCL2	3.1	0.115	2.47	8.60E-03	2.92	2.64E-13	1.8	8.42E-07	0.9	0.844
CXCL3	4.14	0.0351	0.86	6.83E-01	1.66	2.94E-06	1.02	0.00864	1.01	0.901
CXCL6	6.55	0.0281	3.4	6.60E-02	3.56	2.79E-12	2.84	2.11E-06	2.17	0.813
DHRS9	2.63	0.099	-0.36	6.70E-01	2.15	3.50E-12	1.23	1.01E-06	1.24	0.428
DNASE1L3	1.16	0.0177	0	9.98E-01	2.86	9.92E-27	2.19	4.37E-17	1.69	0.214
DSG3	2.32	1.78E-36	0.48	5.05E-01	2.16	7.40E-26	1.99	1.01E-19	1.2	0.106
EPGN	1.01	0.0896	1.64	4.88E-03	4.58	1.14E-20	3.01	1.91E-06	1.62	0.524
EPSTI1	0.13	0.886	1.07	5.68E-02	3	6.14E-19	2.11	2.97E-12	-2.1	0.0359
FGR	0.63	0.25	1.57	4.20E-03	1	1.02E-07	1.37	9.14E-10	0.08	0.984
FOSL1	1.27	0.00472	-0.36	5.77E-01	2.59	2.28E-20	3.33	1.33E-10	3.1	0.229
FPR1	1.11	0.205	1.98	1.28E-06	2.16	2.77E-11	1.27	2.27E-05	3.03	0.253
G0S2	1.01	0.423	1.17	0.0368	1.42	0.00468	1.02	0.0318	-0.44	0.901
GZMA	0.45	0.736	1.14	9.23E-02	3.18	1.67E-19	1.7	3.55E-07	0.85	0.55
ISG15	-0.09	0.905	1.25	6.11E-02	3.58	1.97E-13	1.88	5.01E-07	0.51	0.895
KLK6	2.24	0.0114	1.99	3.20E-02	4.78	2.69E-17	2.74	3.35E-08	0.49	0.91
LCN2	2.14	0.0156	1.07	7.81E-02	5.88	1.06E-19	2.68	2.14E-12	1.73	0.235
MMP9	1.32	0.0568	1.24	1.44E-02	2.97	5.85E-21	2.01	4.85E-11	-0.99	0.614
MX1	-0.24	0.775	1.04	9.56E-02	3.87	1.40E-22	2.25	9.88E-12	1.36	0.146
NABP1	0.54	0.363	1.19	3.32E-02	1.71	4.68E-23	1.22	1.37E-13	0.76	0.45
NEURL3	0.28	0.47	1.06	9.74E-05	1.29	0.000103	1.55	8.76E-07	-0.13	0.998
OLFM4	2.14	0.00101	1.97	1.72E-09	0.91	0.0244	2.77	0.000287	-1	0.89
OLR1	2.65	0.0872	-0.5	8.64E-01	3.18	3.95E-14	1.32	0.00164	-0.69	0.949
PRR9	0.57	0.735	2.03	2.24E-04	2.33	2.58E-05	2.23	0.000772	4.71	0.154
PSMB9	-0.06	0.926	1.1	3.80E-02	1.06	6.11E-13	1.05	2.47E-10	0.29	0.821
SELE	0.44	0.588	1.58	3.37E-03	1.99	1.97E-12	2.41	1.03E-15	2.49	0.35
SELL	0.68	0.275	1.97	4.78E-06	1.23	3.15E-09	1.24	2.03E-06	-0.23	0.945
SPRR1A	1.72	0.024	1.75	4.10E-02	4.22	3.71E-24	3.85	8.74E-15	2.19	0.132
STEAP4	1.12	0.0167	0.74	1.23E-01	2.5	1.70E-18	1.88	1.67E-10	0.65	0.655
TCHH	0.36	0.583	1.24	0.0016	0.99	0.159	1.46	0.0649	5.67	0.102
TNFSF10	0.29	0.68	1.19	1.60E-03	1.87	1.83E-26	1.29	2.82E-13	0.55	0.259
TNFSF14	1.51	0.122	2.17	0.00798	0.63	0.00251	1.29	1.42E-08	-0.74	0.765
TREM1	1.47	0.0836	1.82	1.36E-02	2.54	7.01E-10	1.21	0.00599	-0.69	0.949
TRIM10	1.16	0.0532	0.34	6.55E-01	4.04	1.71E-23	3.63	3.60E-18	2.79	0.232
ZBP1	0.35	0.732	1.31	3.44E-02	3.28	1.93E-12	1.31	3.95E-05	-1.16	0.873

Supplemental Table 7

Downregulated Genes in the *Dsg1*$^{-/-}$ mouse, AD and SAM Syndrome										
Gene	*Dsg1*$^{-/-}$ #1 FC	*Dsg1*$^{-/-}$ #1 FDR	*Dsg1*$^{-/-}$ #2 FC	*Dsg1*$^{-/-}$ #2 FDR	PSO FC	PSO FDR	AD FC	AD FCR	SAM FC	SAM FDR
AFF2	0.26	0.744	-1.13	0.0657	-1.29	2.19E-07	-1.24	7.08E-08	-3.64	0.0141
CNTN6	-1.05	0.0894	-0.3	0.64	-1.17	0.00558	-1.02	0.0151	-4.64	0.0185
ERBB4	-0.13	0.875	-1.81	0.0586	-5.26	2.21E-21	-3.17	8.38E-15	-3.62	0.00405
FRAS1	0.1	0.803	-1.08	0.0692	-1.28	6.49E-08	-1.68	1.63E-12	-3.18	0.0102
PAPPA2	-0.61	0.487	-1.26	0.0442	-2.19	4.02E-07	-1.21	0.000406	-4.83	0.000439
SSC5D	0.15	0.843	-1.01	0.0842	-2.81	1.48E-16	-1.15	4.79E-06	-1.02	0.0805
TENM1	-0.21	0.838	-1.46	0.0723	-2.04	3.59E-20	-1.19	3.39E-09	-1.48	0.00969
ZNF385B	-0.59	0.0583	-1.03	0.0249	-3.47	1.08E-12	-2.14	2.99E-08	-3.64	0.0122

Supplemental Table 8

Downregulated Genes in the *Dsg1*$^{-/-}$ mouse and AD, but not in SAM Syndrome										
Gene	*Dsg1*$^{-/-}$ #1 FC	*Dsg1*$^{-/-}$ #1 FDR	*Dsg1*$^{-/-}$ #2 FC	*Dsg1*$^{-/-}$ #2 FDR	PSO FC	PSO FDR	AD FC	AD FCR	SAM FC	SAM FDR
AIF1L	-1.27	0.00464	-0.49	0.471	-0.77	7.76E-10	-1.16	3.05E-16	-0.33	0.819
BEST3	-0.25	0.761	-1.44	0.0585	-0.39	0.274	-1.46	6.68E-06	0.65	0.949
C5orf46	-1.78	7.93E-07	-1.3	0.567	-3.4	1.66E-21	-3.68	8.30E-24	1.14	0.42
CACNA1H	-0.36	0.426	-1.04	0.0723	-5.14	5.19E-10	-3.51	4.87E-08	-2.65	0.792
ELFN2	0.48	0.686	-1.61	0.0997	-2.69	1.11E-14	-1.71	6.35E-09	0.6	0.957
IGFBP5	-0.09	0.883	-1.37	0.0462	-2.4	1.39E-20	-1.32	6.80E-13	-0.81	0.206
LCE5A	-2.51	0.0718	-2.79	0.099	-2.97	6.84E-18	-4.02	1.84E-24	-0.91	0.612
PLXNA4	0	0.997	-1.17	0.0657	-2.52	9.09E-25	-1.41	8.25E-16	-1.35	0.136
SCUBE1	0.09	0.848	-1.25	0.0574	-3.32	4.31E-15	-2.04	2.22E-10	-1.25	0.896
WNK2	0.25	0.477	-1.34	0.0787	-3.03	1.25E-17	-1.06	5.99E-07	-1.85	0.124

Supplemental Table 9

Primers used for qPCR		
**Gene**	5'	3'
Dsg1a	CAAGGCACTTCTTCCACTGAGA	CGCTGCCTCCCCATGA
Dsg1b	GGAGGCAGTGGAGTTAACAACAC	CGGGTTCCGGTTCGTCTA
Dsg1c	GACCTGCACAGGGACAATCA	GATGTTGTGGGATGTTCAGTAGTTG
Dsg1a	AACCTGCTGGTTGTTGACTC	CGTTGTGGGTTCTCAGTGGA
Dsg1b	AGAACCTGCTGGTTGTTGAC	GGTTCCGGTTCGTCTAAGGG
Dsg1c	CAGGTCAA CTACAAACAAG	GTACCATGATGATTGTCCCTG
Dsg3	CCTGACAGTGTGTCAATGTG	GGCTGAGCTCCTTCGATTCC
Dsg4	GCGGGGATTGATCGGCCACC	CTTGATTCTGCAGTCACATTC
Dsc1	GCTCTGCATTGCTACTGTGC	ACACCTTTTCACCAAGCCGA
Dsc3	ATGGTGGTTCCTGAGTTCCG	TTGAGGCGTGTGTGCATAGT
Pkp1	GCATAACCTCTCCTACCGCC	CCATTGGACATCAGCCCCTT
Pkp2	ACGAAGATGTTCAACGGGCT	CCGAGGCACTCCATTCAGTT
Pkp3	GCAAGCCTGAGACTGGTGTT	TCGCTCATGGAAGGACACTG
Dp	AGCTCGATGGAAAGTCAGCC	GGGAGAGTCTGTCCATCTGGT
Jup	GCTGCCCAGAGTATGATCCC	GGGGTAGTCTCCATCCAGGT
Lor	CTCCTGTGGGTTGTGGAAAGA	TGGAACCACCTCCATAGGAAC
Il1a	GCACCTTACACCTACCAGAGT	AAACTTCTGCCTGACGAGCTT
Il1b	GAAATGCCACCTTTTGACAGTG	TGGATGCTCTCATCAGGACAG
Cxcl1	ACTGCACCCAAACCGAAGTC	TGGGGACACCTTTTAGCATCTT
Cxcl2	CCAACCACCAGGCTACAGG	GCGTCACACTCAAGCTCTG
s100a8	CCTTTGTCAGCTCCGTCTTCA	TCCAGTTCAGACGGCATTGT
s100a9	GCACAGTTGGCAACCTTTATG	TGATTGTCCTGGTTTGTGTCC
Flg1	ATGTCCGCTCTCCTGGAAAG	TGGATTCTTCAAGACTGCCTGTA
Flg2	CTAGAGGGCATGAGTGTAGTCA	CAAGACTGGACAGTTGGCTGG
Ivl	ATGTCCCATCAACACACACTG	TGGAGTTGGTTGCTTTGCTTG
Tgm1	TCTGGGCTCGTTGTTGTGG	AACCAGCATTCCCTCTCGGA
Cdsn	TTGCTGATGGCCGGTCTTATT	GCCAGTCTTTCCAATGAGACAAG
Cdh1	CAGGTCTCCTCATGGCTTTGC	CTTCCGAAAAGAAGGCTGTCC
Cdnnb1	ATGGAGCCGGACAGAAAAGC	CTTGCCACTCAGGGAAGGA
Cdnna1	AAGTCTGGAGATTAGGACTCTGG	ACGGCCTCTCTTTTTATTAGACG
Cdh3	CTGGAGCCGAGCCAAGTTC	GGAGTGCATCGCATCCTTCC
Krt1	TGGGAGATTTTCAGGAGGAGG	GCCACACTCTTGGAGATGCTC
Krt10	CGAAGAGCTGGCCTACCTAAA	GGGCAGCGTTCATTTCCAC
Krt14	AGCGGCAAGAGTGAGATTTCT	CCTCCAGGTTATTCTCCAGGG
Krt5	TCTGCCATCACCCCATCTGT	CCTCCGCCAGAACTGTAGGA

Supplemental References

1. Dusek RL, et al. The differentiation-dependent desmosomal cadherin desmoglein 1 is a novel caspase-3 target that regulates apoptosis in keratinocytes. *J Biol Chem.* 2006;281(6):3614-3624.
2. Swindell WR, et al. Genome-wide expression profiling of five mouse models identifies similarities and differences with human psoriasis. *PLoS One.* 2011;6(4):e18266.
3. Dobin A, et al. Star: Ultrafast universal rna-seq aligner. *Bioinformatics.* 2013;29(1):15-21.
4. Anders S, et al. Htseq--a python framework to work with high-throughput sequencing data. *Bioinformatics (Oxford, England).* 2015;31(2):166-169.
5. Love MI, et al. Moderated estimation of fold change and dispersion for rna-seq data with deseq2. *Genome Biol.* 2014;15(12):550.
6. Tsoi LC, et al. Atopic dermatitis is an il-13-dominant disease with greater molecular heterogeneity compared to psoriasis. *J Invest Dermatol.* 2019;139(7):1480-1489.
7. Scothern A, and Garrod D. Visualization of desmosomes in the electron microscope. *Methods Cell Biol.* 2008;88:347-366.

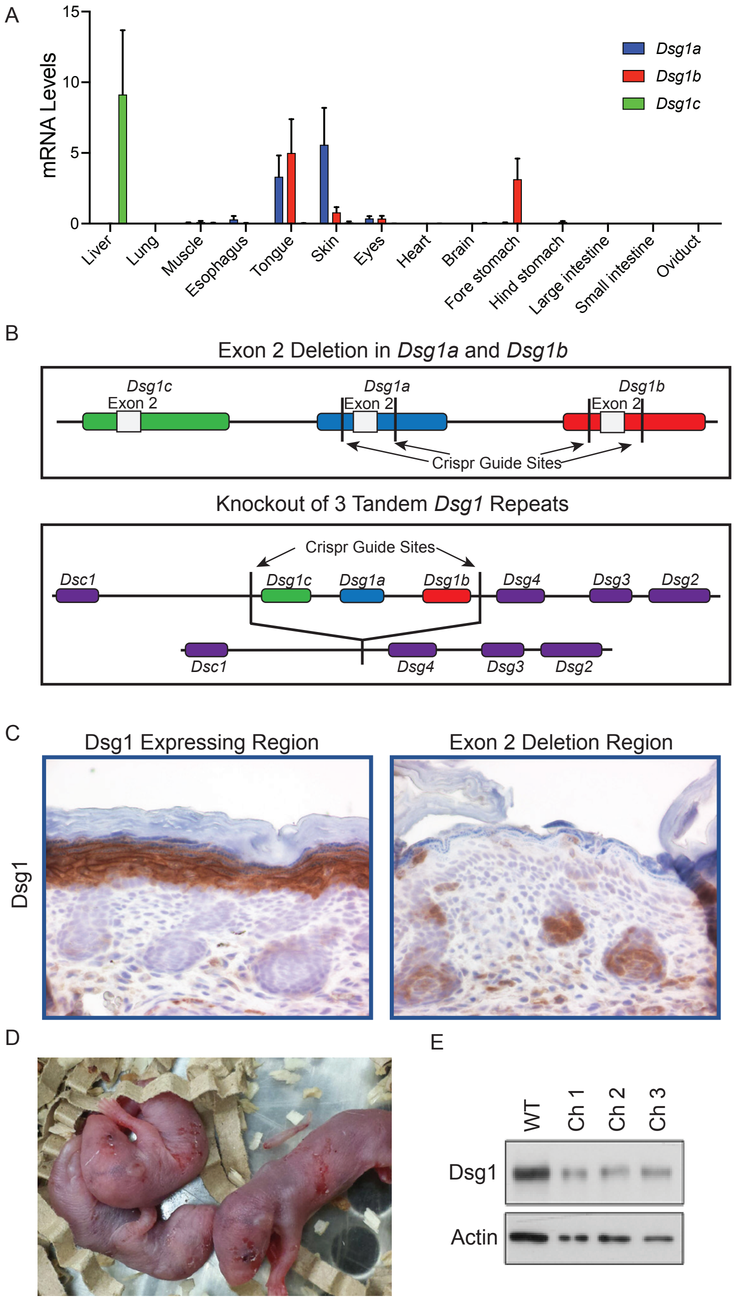

Supplemental Figure 1. Exon 2 deletion in mice causes perinatal lethality and reduced Dsg1 in blistering areas. (A) Real-time qRT-PCR from RNA isolated from mouse organs demonstrating the expression of *Dsg1a*, *Dsg1b*, and *Dsg1c*. (data represent mean ± SEM, n = 3). **(B)** Schematic representing the cadherin gene cluster in mice and describing the two knockout strategies pursued. **(C)** Immunostaining for Dsg1 in Dsg1 exon 2 deletion chimeras showing areas expressing Dsg1 and areas with exon 2 deletion (40x magnification, n = 3 Dsg1 exon 2 deletion chimeras). **(D)** Images of newborn chimeras demonstrating the presence of skin blisters. **(E)** Immunoblot for Dsg1 in skin samples from 1 *Dsg1*$^{+/+}$ and 3 *Dsg1* exon 2 deletion chimeric animals.

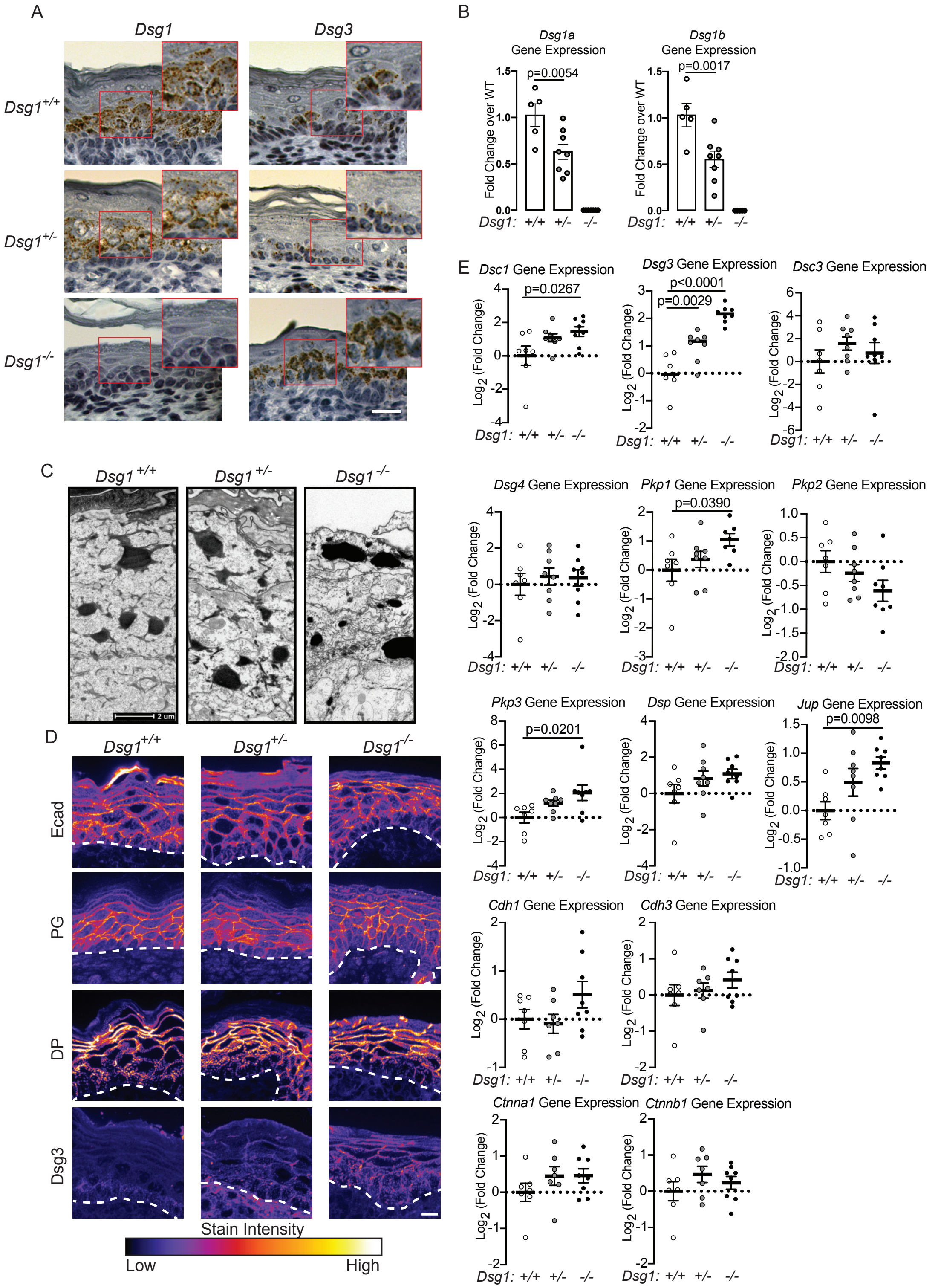

Supplemental Figure 2. Dsg1 loss perturbs normal keratinocyte adhesion and expression of adhesion molecules. (A) RNAscope showing *Dsg1* and *Dsg3* gene expression in E18.5 skin. Scale bar = 20 µm **(B)** Gene expression for *Dsg1a* and *Dsg1b* in E18.5 skin was calculated using the ΔΔCT method, normalizing to GAPDH and then the $Dsg1^{+/+}$ mouse (data represent mean ± SEM, n = 5-8/genotype). **(C)** Transmission electron microscope images of E18.5 mouse skin. Scale bar = 2 µm (n = 3/genotype). **(D)** Immunostaining for E-cadherin (Ecad), plakoglobin (PG), desmoplakin (DP) and desmoglein 3 (Dsg3) in skin from E18.5 mice. Scale bar = 20 µm (data represent mean ± SEM, n = 3-13/genotype). **(E)** Gene expression for cadherin and cadherin associated genes in skin from E18.5 mice. Fold change in gene expression was calculated using the ΔΔCT method, normalizing to GAPDH and then the $Dsg1^{+/+}$ mouse (data represent mean ± SEM, n = 7-8/genotype). Statistical significance for **(B and E)** was determined using one-way ANOVA with a Tukey correction for multiple comparisons.

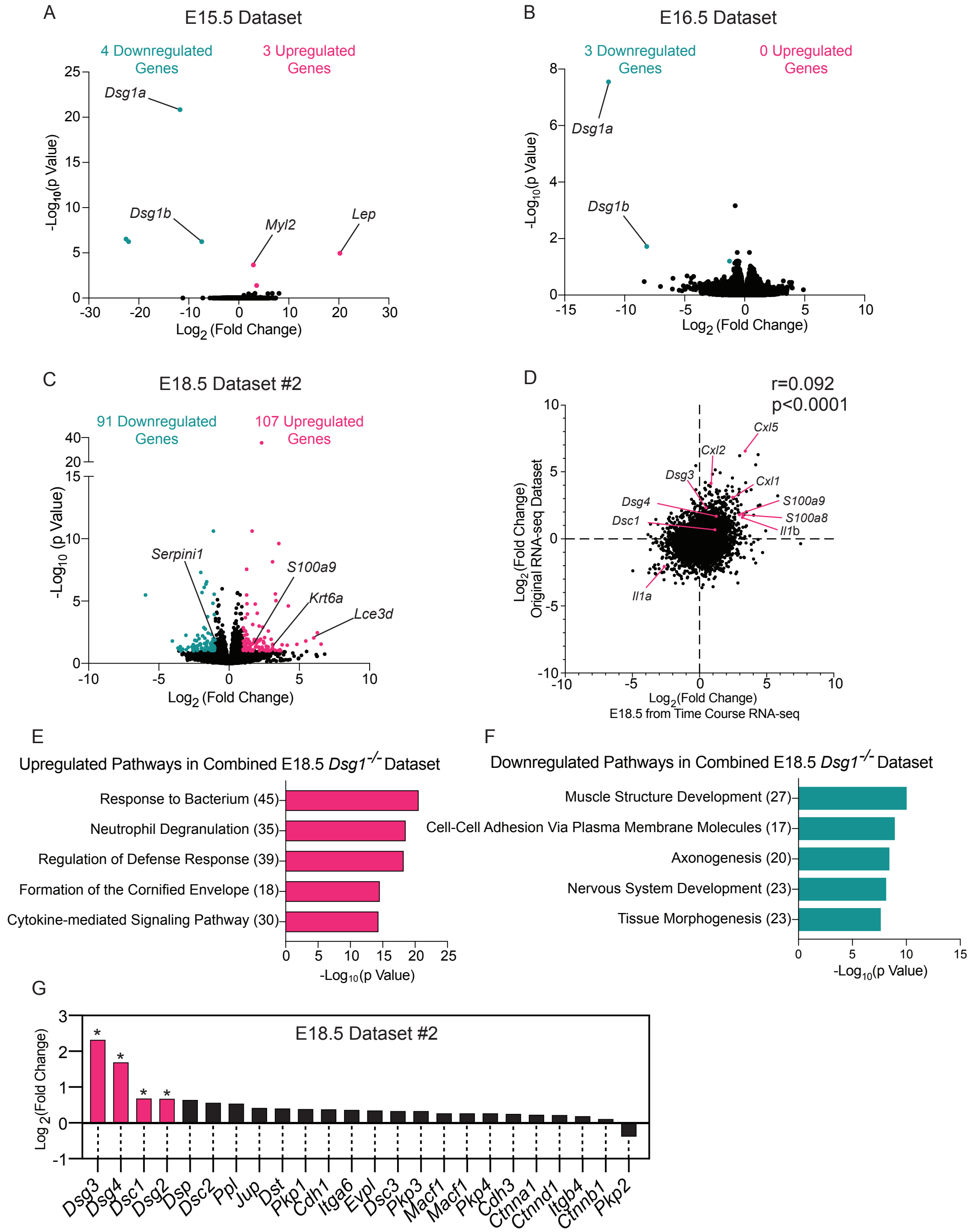

Supplemental Figure 3. RNA-seq analysis of embryonic timepoints and comparison of two *Dsg1*$^{-/-}$ E18.5 datasets showing a high degree of similarity in affected pathways. (A, B) Volcano plot of upregulated and downregulated genes from RNA-seq analysis performed on **(A)** E15.5 skin and **(B)** E16.5 skin. False Discovery Rate (FDR) ≤ 0.1 and |log$_2$ (Fold Change (FC))| ≥ 1 considered significant. (n = 4/genotype). **(C)** Volcano plot of upregulated and downregulated genes from RNA-seq analysis performed on E18.5 dataset #2 (n = 5/genotype). **(D)** Scatter plot showing the concordance between the 1st and 2nd RNA-seq datasets from E18.5 *Dsg1*$^{-/-}$ mice. **(E)** Functional enrichment analysis performed using genes upregulated in either of the *Dsg1*$^{-/-}$ mouse RNA-seq datasets. Values in parentheses represent number of genes associated with each pathway. **(F)** Functional enrichment analysis performed using significantly downregulated genes in either of the *Dsg1*$^{-/-}$ mouse RNA-seq datasets. Values in parentheses represent number of genes associated with each pathway. **(G)** mRNA expression levels for proteins that make up desmosomes, adherens junctions, and hemidesmosomes from the E18.5 time course RNA-seq dataset #2 (* FDR < 0.1).

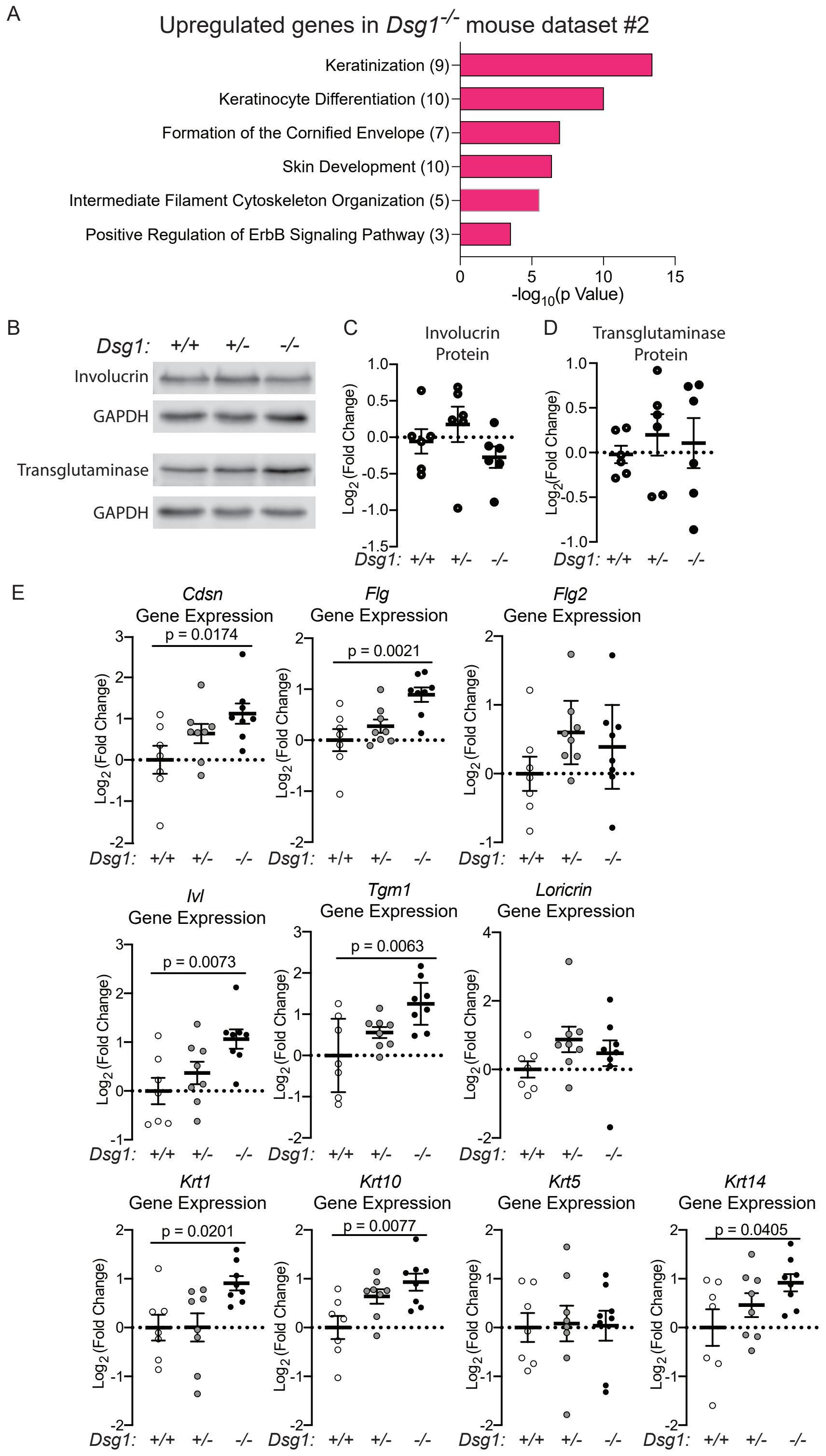

Supplemental Figure 4. Dsg1 knockout disrupts normal keratinocyte differentiation. (A) Functional enrichment analysis on genes significantly upregulated in the E18.5 *Dsg1*$^{-/-}$ mouse RNA-seq dataset #2. Value in the parentheses represent the number of genes associated with each pathway. **(B)** Immunoblot for involucrin and transglutaminase in protein extracts from E18.5 mouse skin. GAPDH was used as a loading control. **(C)** Quantification of involucrin protein from immunoblot. Densitometry values were normalized to the *Dsg1*$^{+/+}$ samples and GAPDH (data represent mean ± SEM, n = 6/genotype). **(D)** Quantification of transglutaminase protein from immunoblot. Densitometry values were normalized to the *Dsg1*$^{+/+}$ samples and GAPDH (data represent mean ± SEM, n = 6/genotype). **(E)** Gene expression for genes expressed in keratinocyte differentiation pathways (n = 7-8/genotype). Fold change in gene expression was calculated using the ΔΔCT method, normalizing to GAPDH and then the *Dsg1*$^{+/+}$ mouse (data represent mean ± SEM, n = 7-8/genotype). Statistical significance for **(C, D, and E)** was determined using one-way ANOVA with a Tukey correction for multiple comparisons.

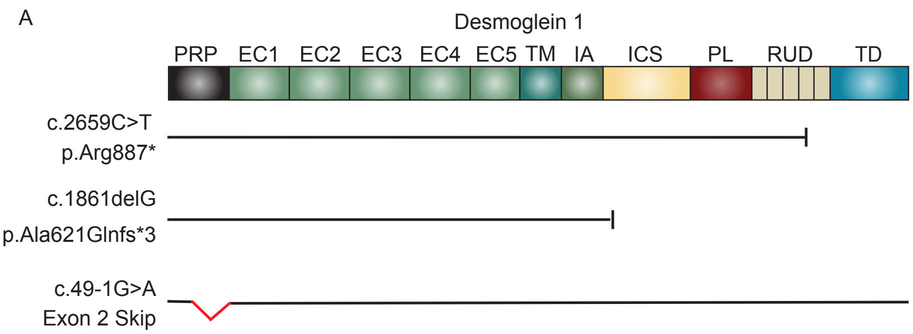

Supplemental Figure 5. Dsg1 domains and associated mutations in SAM syndrome patients. (A) Schematic showing protein domains for Dsg1 and mutations associated with patients from SAM syndrome used in this manuscript. PRP, signal peptide and proprotein; EC, extracellular; TM, transmembrane; IA, intracellular anchor; ICS, intracellular cadherin-like sequence; PL, intracellular proline-rich linker; RUD, repeat unit domain; TD, desmoglein specific terminal domain.

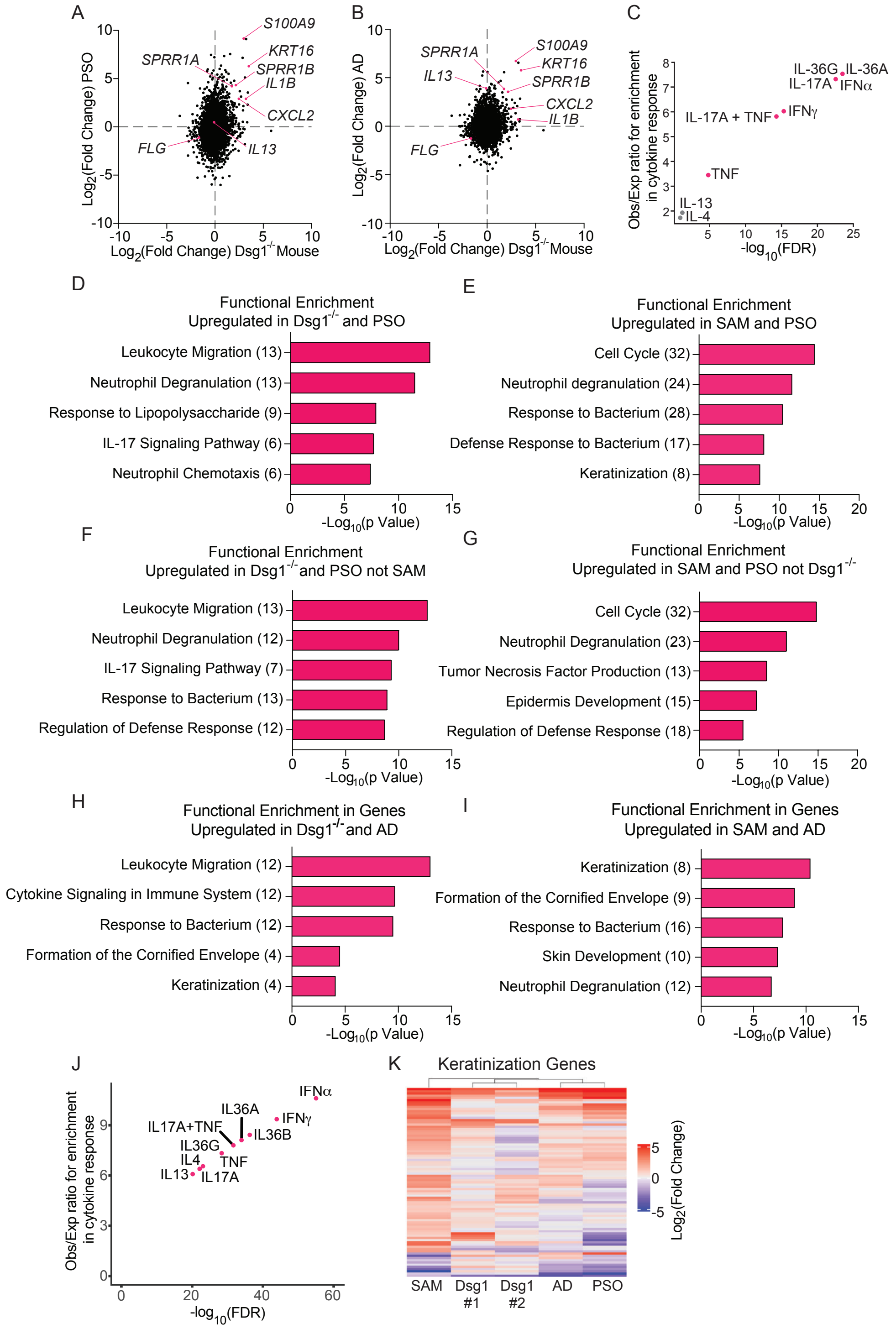

Supplemental Figure 6. Similarities among *Dsg1$^{-/-}$* mice, SAM, PSO and AD. (A) Scatter plot showing concordance between genes in PSO patients and the E18.5 RNA-seq dataset #1 from the *Dsg1$^{-/-}$* mouse. **(B)** Scatter plot showing concordance between genes in AD patients and the E18.5 RNA-seq dataset #1 from the *Dsg1$^{-/-}$* mouse. **(C)** Upregulated gene signatures from PSO RNA-seq compared with those from keratinocytes treated with cytokines in culture. Statistically significant similarities are indicated in red (adj p value < 0.05). **(D-I)** Functional enrichment analysis in genes upregulated in **(D)** E18.5 *Dsg1$^{-/-}$* skin and PSO, **(E)** SAM and PSO, **(F)** E18.5 *Dsg1$^{-/-}$* skin and PSO excluding genes also upregulated in SAM, **(G)** SAM and PSO excluding genes also upregulated in *Dsg1$^{-/-}$* skin, **(H)** E18.5 *Dsg1$^{-/-}$* skin and AD, **(I)** SAM and AD. **(J)** Comparison between RNA-seq datasets of cytokine stimulated keratinocytes and AD patients. Data are plotted as mentioned above in **(C)**. **(K)** Heatmap showing expression levels of genes associated with the keratinization gene ontology pathway in both E18.5 datasets as well as the SAM, PSO and AD datasets.

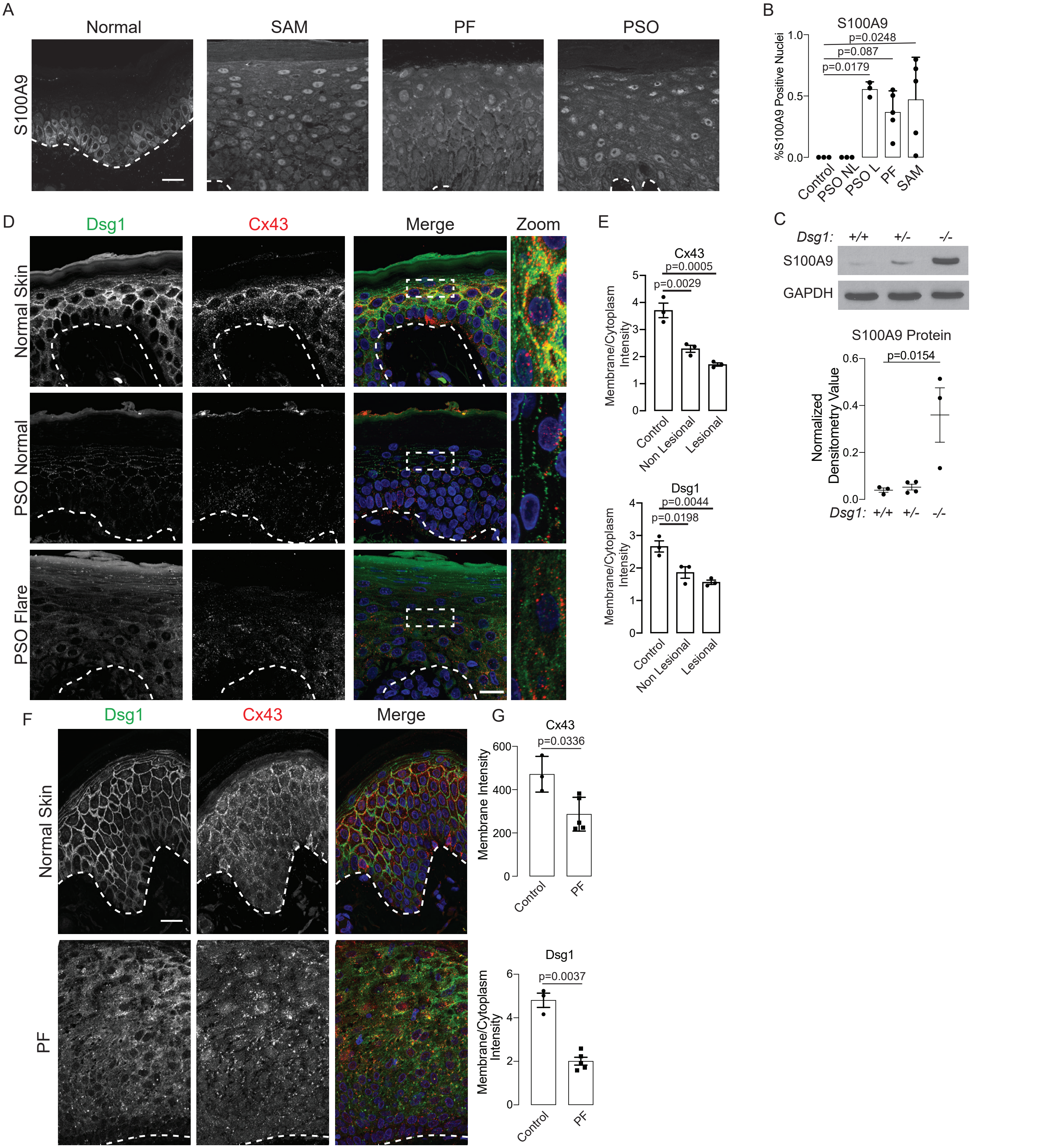

Supplemental Figure 7. Loss of Dsg1 is associated with alterations in S100A9 and other junctional proteins across disease types. (A) Immunostaining for S100A9 in control, SAM Syndrome, PF and PSO patients. Scale bar = 20 µm. **(B)** Quantification of % number of nuclei positive for S100A9 (data represent mean ± SEM, n = 3-5/group). **(C)** Immunoblot for S100A9 in E18.5 mouse skin. GAPDH was used as loading control. Densitometry values were normalized to GAPDH (data represent mean ± SEM, n = 3-4/genotype). **(D)** Immunostaining for Dsg1 and Cx43 in normal skin, non-lesional skin and lesional skin from PSO patients. Scale bar = 20 µm. **(E)** Quantification of Cx43 and Dsg1 in immunostained samples expressed as membrane intensity over cytoplasmic intensity (data represent mean ± SEM, n = 3). **(F)** Immunostaining for Dsg1 and Cx43 in normal skin and skin from PF patients. Scale bar = 20 µm. **(G)** Quantification of Cx43 and Dsg1 immunostained samples expressed as membrane intensity or membrane intensity over cytoplasmic intensity in PF and control samples (data represent mean ± SEM, n = 3-5). Statistical significance for **(B, C, and E)** were determined using one-way ANOVA with a Tukey correction for multiple comparisons. Statistical significance for **(G)** was determined by Student's t-test.

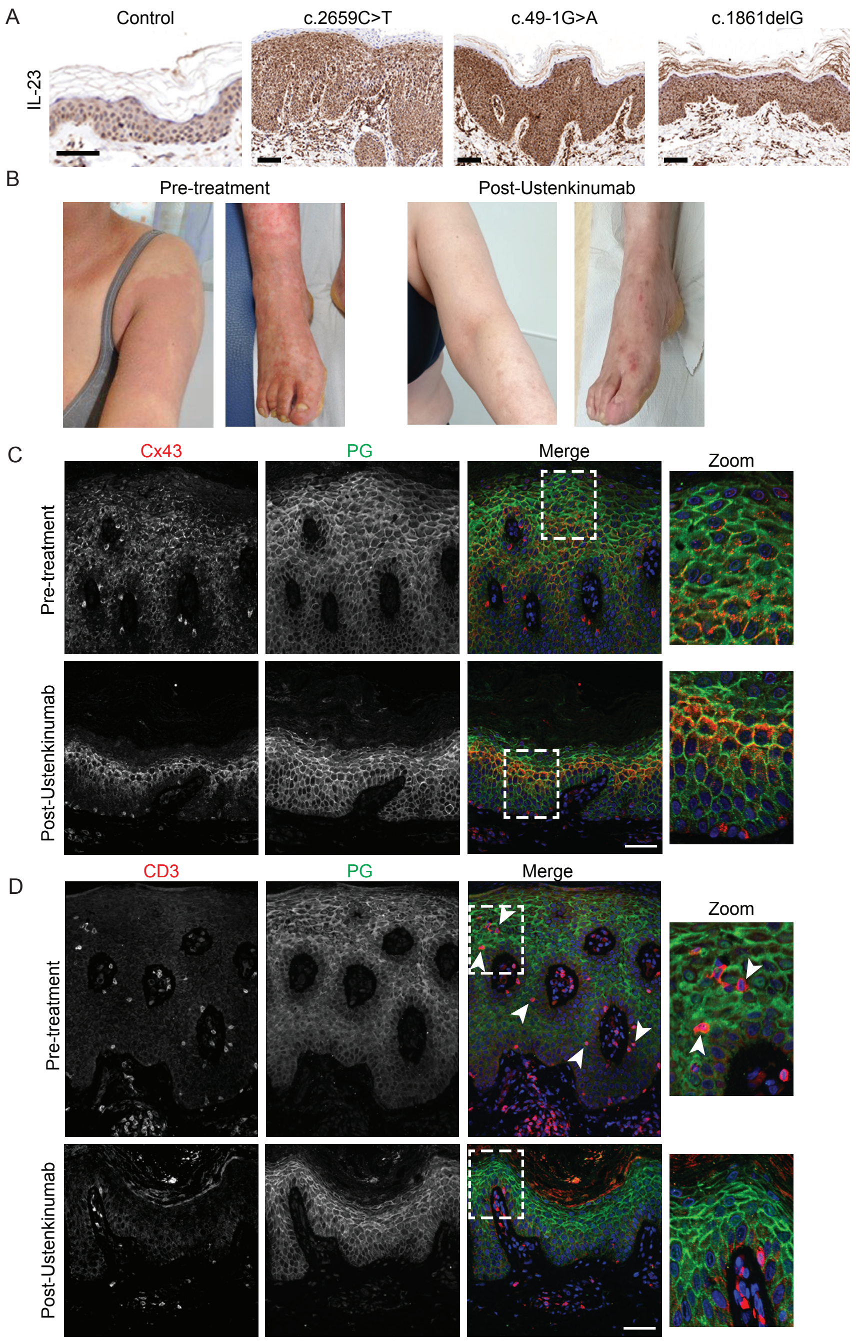

Supplemental Figure 8. IL-23 is upregulated in the lesional skin in SAM Syndrome patients. (A) Immunohistochemical staining for IL-23 in control and SAM Syndrome patients. Mutation status of the patient is noted above each image. Scale bars = 100 μm (n = 1/mutation). **(B)** Clinical photos of the sibling/patient #2 with SAM Syndrome before and after 4 weeks of treatment with the Il-12/IL-23 blocking antibody ustekinumab (n = 1 patient). **(C)** Immunostaining for Cx43 and plakoglobin (PG) in skin biopsies collected before and after 12 weeks of treatment with ustekinumab in patient #1 showing restoration of Cx43 staining following treatment (n = 1 patient, scale bar = 50 μm). **(D)** Immunostaining for CD3 and PG in skin biopsies collected pre-treatment and after 12 weeks of treatment with ustekinumab in patient #1 showing reduced CD3-positive immune cells following treatment. Arrowheads indicate CD3 positive cells infiltrating the epidermis in the pre-treatment SAM Syndrome skin (n = 1 patient, scale bar = 50 μm).

Figure 1B Full Immunoblots

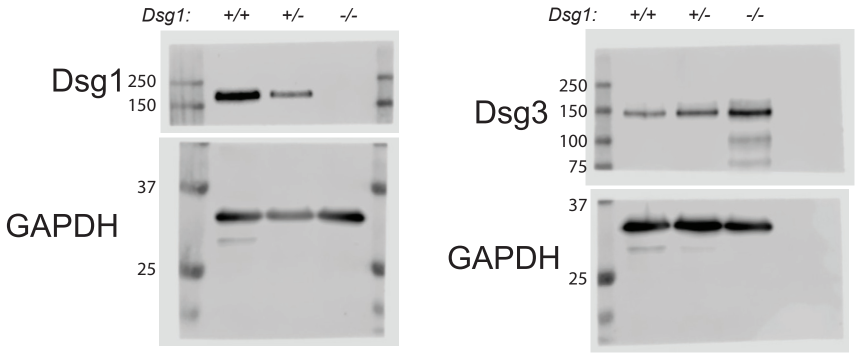

Figure 2B Full Immunoblots

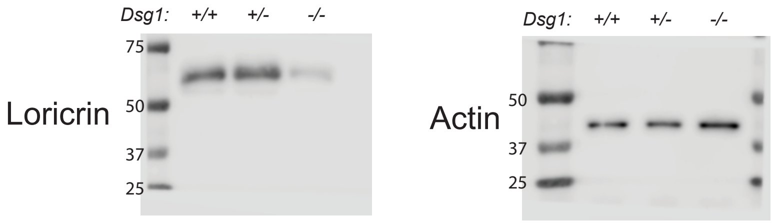

Supplemental Figure 4B Full Immunoblots

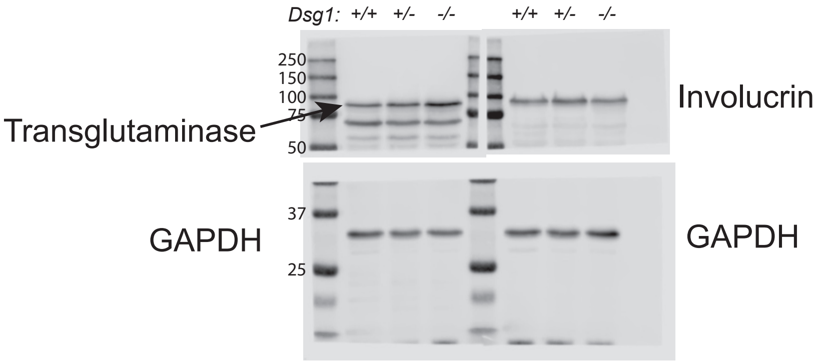

Supplemental Figure 7C Full Immunoblots

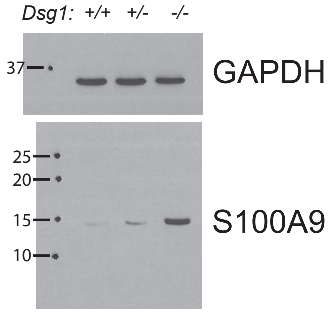

Supplement: Supplemental data [file jci-132-144363-s068.pdf]
